# Unusual Polycyclic Fused Product by Oxidative Enzymatic Dimerisation of 5-methylpyrogallol Catalysed by Horseradish Peroxidase/H_2_O_2_

**DOI:** 10.3390/molecules23102619

**Published:** 2018-10-12

**Authors:** Hélène Bouges, Kevin Calabro, Olivier P. Thomas, Sylvain Antoniotti

**Affiliations:** 1 Institut de Chimie de Nice, Université Cote d’Azur, CNRS, Parc Valrose, 06108 Nice CEDEX 2, France; helene.bouges@unice.fr; 2 Marine Biodiscovery, School of Chemistry and Ryan Institute, National University of Ireland Galway, University Road, H91 TK33 Galway, Ireland; kevin.calabro@nuigalway.ie (K.C.); olivier.thomas@nuigalway.ie (O.P.T.)

**Keywords:** cage structure, biocatalysis, phenolics, molecular complexity, sustainable chemistry

## Abstract

During investigations on the peroxidase-catalysed oxidation of polyhydroxylated monoaromatic substrates such as 5-methylpyrogallol, we observed a spectacular dimerisation proceeding by dearomatisation in contrast with most common reaction patterns involving phenolics oxidation and dimerization. A tetracyclic fused product featuring an unusual 2-oxatetracyclo [6.3.1.0^1,6^.0^4,12^] dodecan-3-one core was obtained and characterized by combined NMR techniques and high resolution mass spectroscopy (HRMS). This is an example of a spontaneous cascade triggered by a simple enzymatic reaction that could provide new options for biosynthetic hypothesis and a synthetic method to access this complex core in one operation.

## 1. Introduction

The potential of enzymatic reactions to achieve the synthesis of complex molecules is still largely underexplored despite a broad array of applications in bioengineering. Such green transformations might yield to a useful chemical diversity already present in nature and potentially offering interesting bioactivities. Peroxidases from soybean (SBP) or horseradish (HRP) are known to catalyse the one-electron oxidation of phenol to the corresponding phenoxy radical in the presence of hydrogen peroxide [1]. Since the generated phenoxy radicals can quickly undergo non-enzymatic coupling in solution through C-O or C-C bonding leading to dimers and subsequently higher order oligomers, peroxidase chemistry has been widely used for phenolics manipulation, mostly for polymerisation purposes [2]. In the case of substituted phenolic substrates, electronic and steric effects significantly influence the course of the reaction and various patterns of dimeric coupling can be observed, usually following the C-C *ortho* coupling [3].

**Scheme 1:**
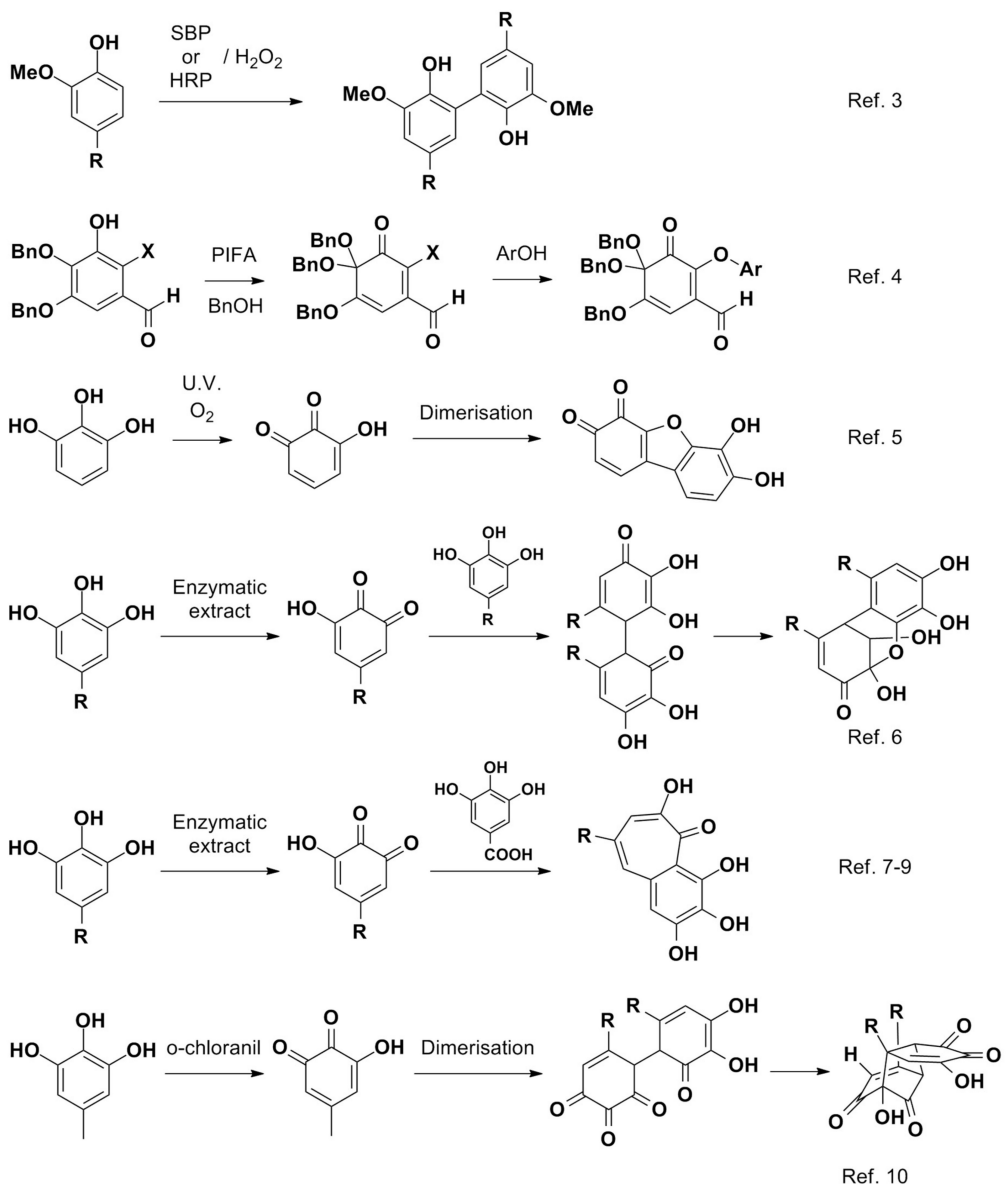
Most common oxidation products of mono-aromatic phenols.

Substituted phenol derivatives have also been showed to yield reactive *ortho*-quinones under oxidative conditions, which underwent oxa-Michael addition, elimination and reductive aromatization with other phenolics [4]. In regards of phenolics containing more than two hydroxyl groups, different oxidation patterns and reaction outcomes were reported. For example, pyrogallol dimerised under UV-irradiation and O_2_ to yield dibenzofuran derivatives and higher order oligomers [5]. Closely related to pyrogallol, gallate esters led to catechin oligomers when submitted to enzymatic oxidation [6]. The spontaneous oxidation of quercetin under air yielded theaflavines following another reaction pathway [7,8]. This oxidation of quercetin to theaflavines can also be catalysed by laccases [9]. Oxidative dimerisation of methyl gallate in the presence of *o*-chloranil, a tetrachloro-*para*-quinone, delivered dimethyl dehydrohexahydroxydiphenoates after oxidation of gallate to the corresponding *ortho*-quinone and subsequent C-C bond forming processes (Scheme 1) [10].

Interestingly, no study has been reported to our best knowledge on the enzymatic reactivity of other simple mono-substituted pyrogallol derivatives.

## 2. Results and Discussion

**Scheme 2:**
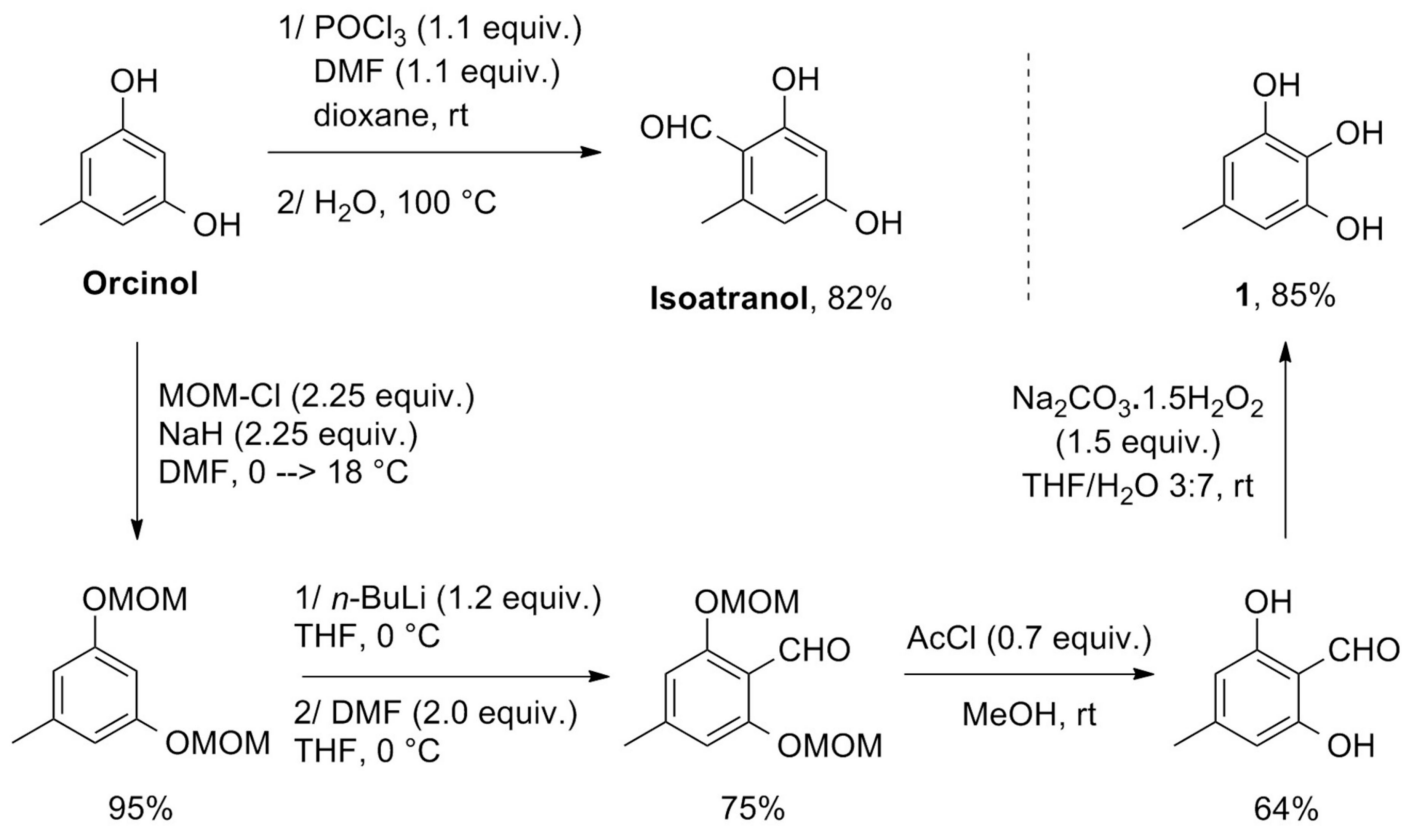
Synthesis of 5-methylpyrogallol **1** from orcinol.

We decided to inspect the behaviour of polyhydroxylated benzenic structures such as 5-methylbenzene-1,2,3-triol (5-methylpyrogallol) in the presence of an isolated oxidative enzyme such as HRP in the presence of H_2_O_2_ as oxidant. The synthesis of 5-methylpyrogallol **1** was achieved in four steps from commercially available orcinol by MOM-protection of both hydroxyl groups, *ortho*-lithiation and formylation with DMF, deprotection and Dakin oxidation following literature procedures (Scheme 2) [11,12]. It is worth noting that standard formylation procedure by POCl_3_/DMF led regioselectively to isoatranol instead of **1**.

Testing of HRP-catalysed oxidation was conducted with **1** in the presence of H_2_O_2_ at pH 9 varying enzyme loading and peroxide stoichiometry, along with relevant control experiments (Table 1). In a representative reaction, the substrate **1** is dissolved in an aqueous sodium carbonate buffer solution at pH 9, containing HRP (1% *w*/*w*). The reactor is then wrapped with an aluminium foil and an aqueous solution of H_2_O_2_ is added slowly to the reaction mixture. The resulting solution is stirred at room temperature for 6 h and, after completion of the reaction, a diluted aqueous solution of HCl is added to reach a final pH of 4 and the resulting aqueous phase is finally extracted with EtOAc.

In the absence of H_2_O_2_ and regardless of the presence of HRP, the starting material was recovered unchanged almost quantitatively (entries 1, 2). In the presence of 2 equiv. of H_2_O_2_ without enzyme, only 34% of starting material was recovered upon extraction with EtOAc (entry 3). Inspection of the aqueous phase revealed the formation of a mixture of various products that could not be identified. In the presence of both H_2_O_2_ and HRP, the starting material was totally converted after 6 h and no product was obtained after extraction of the aqueous reaction mixture by EtOAc (entry 4). Upon evaporation of the water phase, product **2** was obtained as a white solid in 89% yield and submitted to a standard acetylation protocol leading to **2′**. Variation of HRP/H_2_O_2_ equivalents did not show significant improvements for the conversion/yield (entries 5–10). Upon GC-MS-QTOF analysis, starting with a *m*/*z* of 419.0950, we identified a formula of C_20_H_19_O_10_ for [M + H^+^] (419.0978, Δ 6.6 ppm) for **2′**. While the dimeric nature of the product was concluded from HRMS and ^13^C NMR analyses, the structure of the dimer **2′** did not match with usual oxidative products in peroxidase chemistry and we therefore embarked into a full structure elucidation of the product **2′** (Table 2). Three acetyl groups were first evidenced by the presence of three singlets at *δ*_H_ 2.05 (3H), 2.10 (3H) and 2.23 (3H), while another methyl was associated with the signal at *δ*_H_ 1.67 (s, 3H, H-14) and other signals at *δ*_H_ 5.16 (d, *J* = 2 Hz, 1H, H-15b), 5.26 (d, *J* = 2 Hz, 1H, H-15a) and *δ*_C_ 120.9 (CH_2_, C-15) revealed the presence of an exomethylene olefinic group. The conjugation of this first olefin with a second one was inferred from the presence of other olefinic signals at *δ*_H_ 6.12 (s, 1H, H-10) and *δ*_C_ 118.5 (CH, C-10) HMBC correlated with H_2_-15. The dearomatisation of the first monomer **1** was then evidenced through the key H-10/C-1, H_2_-15/C-8 HMBC correlations. Additional HMBC correlations from the signal at *δ*_H_ 3.86 (s, 1H, H-8) like H-8/C-1 allowed closure of the ring of the first monomer with oxygen atoms connected to C-11, C-1 and C-12 due to characteristic chemical shifts. Further H-8/C-13 and H-8/C-7 correlation allowed the connection of the first monomer with the second one, C-13 corresponding to an ester function due to characteristic chemical shifts.

H-6/C-13, C-7, C-5 and C-14 together with H-4/C-6 and C-5 HMBC correlations from the signal at *δ*_H_ 3.86 (s, 1H, H-6) and 3.62 (s, 1H, H-4) finished to establish the arrangement of the second monomer therefore evidencing an oxidation at positions C-3 and C-13 followed by a cleavage into two carboxylic functions at these positions. Even if no HMBC correlation enabled location of C-3 in the chain, its presence was deduced from characteristic chemical shifts and logics into the structure of the second monomer. Connections with the first monomer were inferred from additional HMBC correlations from the methines signals. The three acetyloxy were located at positions C-11, C-12 and C-7 due to final HMBC correlations. No ambiguity was left on the final structure of **2′** using HRMS data and the molecular formula. To the best of our knowledge, this complex cage polycyclic structure is unprecedented in the literature and it shows some similarities with vinigrol [13] epicolactone [14] jiadifenolide [15] or (−)-11-*O*-debenzoyltashironin [16], which require multistep synthesis to be built. Several attempts to obtain monocrystals of **2** for X-ray spectroscopy were unsuccessful.

The structure of **2** before acetylation was slightly different from the one of **2′**. NMR analysis was performed in D_2_O and the spectra featured some significant differences (Table 3). No exo-methylene and of course no acetyl groups were found in this structure and only one olefinic signal was observed at a relatively low field (6.45 ppm). Consequently, two methyl groups could be identified. The three saturated and fused methines of **2′** were still present in **2**. The ^13^C-NMR spectra featured a new signal at 197.2 ppm suggesting a conjugated carbonyl group, and two signals at 178.0 and 171.5 ppm eligible to be those of carbonyl groups of an acid and an ester function, respectively.

HPLC-MS was performed and allowed obtaining a spectrum of **2** by ESI. In negative mode, [M − H]^−^ ion was observed at *m*/*z* 309, although weak, together with strong [2M − H]^−^ at *m*/*z* 619 and [2M − 2H + Na]^−^ at *m*/*z* 641. Under enantioselective SFC and upon polarimetry measurements, product **2** appeared as a racemate. However, the diastereomeric control during the overall process is impressive since only one diastereomer was formed.

**Scheme 3:**
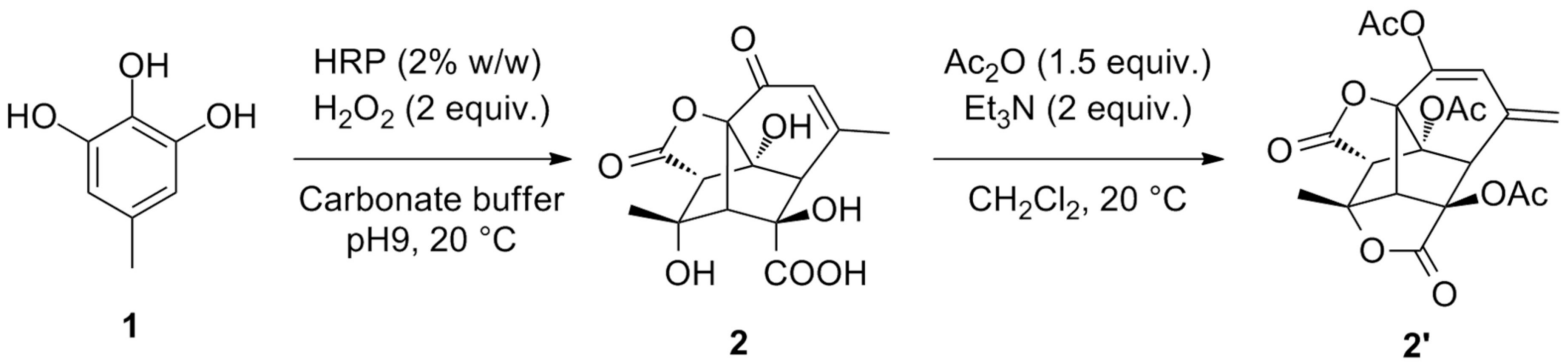
Synthesis of dimers **2** and **2′** from 5-methylpyrogallol **1**.

Under acetylation conditions, the α,β-unsaturated carbonyl motif of **2** was thus enolised to the corresponding dienol and trapped as an acetyl dienolate. In parallel, the γ-hydroxyacid underwent lactonisation to the corresponding γ-lactone fusing a fourth cycle to the existing structure, and the two tertiary alcohol functions were acetylated (Scheme 3).

**Scheme 4:**
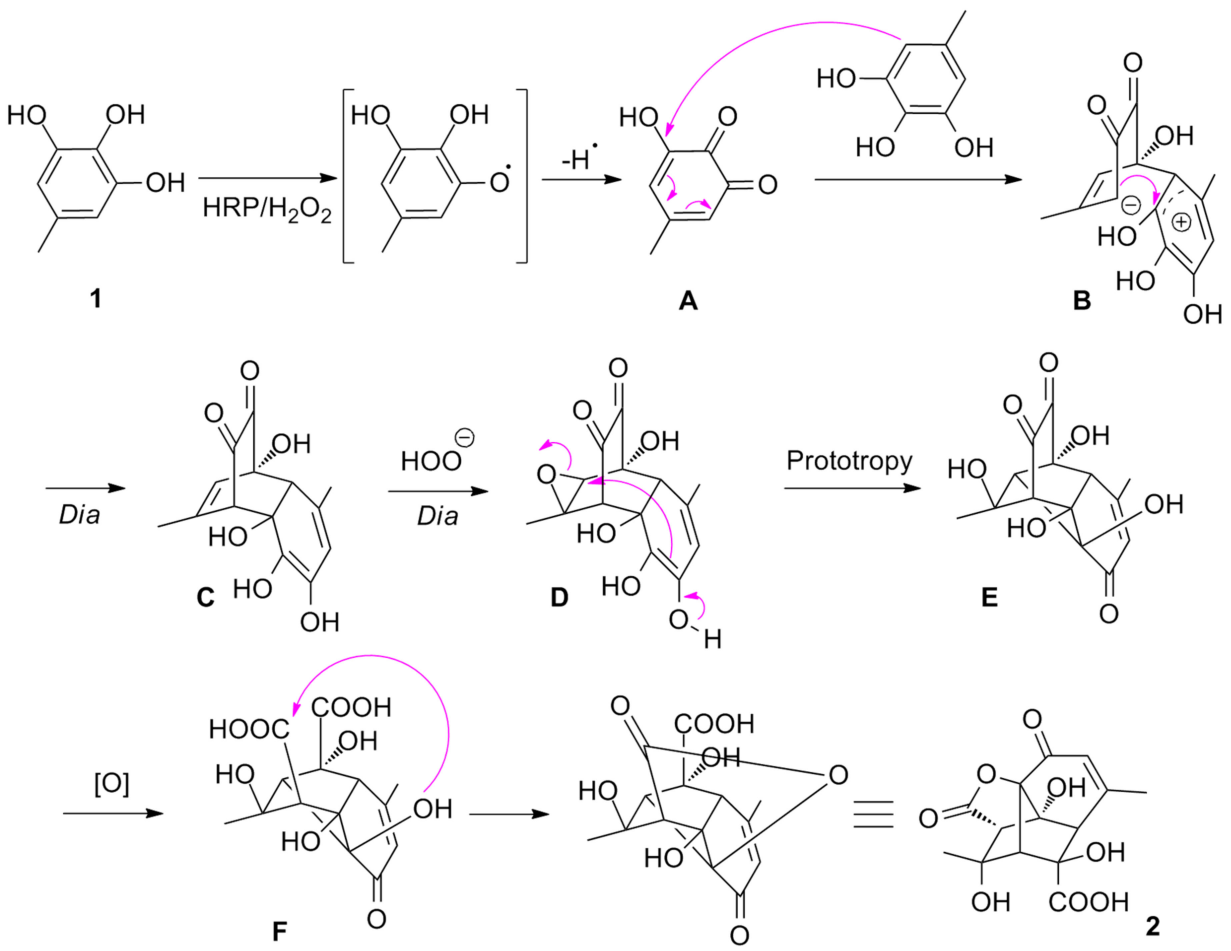
Proposed mechanism to account for the formation of dimer **2** from 5-methylpyrogallol **1**.

From a mechanistic point of view, we propose a series of oxidation/cyclisation to convert **1** into **2** (Scheme 4). Inspired by the reactivity of gallate derivatives in oxidative conditions [10], we hypothesised that substrate **1** could be slowly oxidised into the corresponding *o*-quinone **A** by HRP/H_2_O_2_, which could quickly react with unconverted **1** to yield intermediate **B** upon a vinylogous Michael addition followed ring closure by attack of the resulting enolate towards the arenium intermediate to form **C** in a distereoselective fashion. Epoxidation of the trisubstituted double bond by H_2_O_2_ present in excess in basic medium could then lead to **D** diastereoselectively. A subsequent regio- and stereoselective nucleophilic attack of an enol on the epoxide would then deliver **E** after prototropy. Upon oxidative cleavage of the α-diketone motif to **F** and subsequent lactonisation, product **2** would then be obtained, the oxidative cleavage possibly taking place earlier in the sequence.

## 3. Materials and Methods

^1^H NMR and ^13^C NMR spectra were recorded on a Bruker (Billerica, MA, USA) AC (200 and 400 MHz). ^1^H NMR spectra are reported as follows: Chemical shifts in ppm (δ) relative to the chemical shift of TMS at 0 ppm, integration, multiplicities (s = singlet, d = doublet, t = triplet, q = quartet, m = multiplet and br = broad), and coupling constants (Hz). ^13^C NMR spectra chemical shifts are reported in ppm (δ) relative to CDCl_3_ at 77.16 ppm. Identity was assessed by comparison with data of authentic samples or literature data for **1** and its synthetic intermediates.

Column chromatography was carried out on silica gel (spherical 15–30 µm, neutral, 63–200 µm, Geduran Si 60, Merck KGaA, Darmstadt, Germany).

GC-TCD analyses were carried out using a Shimadzu (Kyoto, Japan) QP2010 plus gas chromatograph, under the following operation conditions: Vector gas, He; injector temperature, 250 °C; detector temperature, 210 °C at 60 mA; split ratio, 1/20; total flow, 22.5 mL min^−1^; Phenomenex (Torrance, CA, USA) Zebron ZB5MS column, polydimethylsiloxane (10 m, inside diameter 0.10 mm, film thickness 0.10 μm); temperature program, 80–200 °C at 10 °C min^−1^ and 200 °C for 8 min.

GC/MS analyses were performed by using a Shimadzu QP2010 gas chromatograph (conditions: carrier gas, He; injector and detector temperatures, 250 °C; injected volume, 0.5 μL; split ratio, 1/100; pressure, 180 kPa; SLB-5 ms capillary column (thickness: 0.25 mm, length: 30 m, inside diameter: 0.25 mm); temperature program, 60–315 °C at 10 °C min^−1^, and 10 min at 315 °C) coupled to a mass selective detector. Mass spectra were obtained by electron ionisation at 70 eV, *m*/*z* 35–400, source temperature 250 °C; only the most abundant ions are given.

High resolution mass spectrometry (HRMS) was performed at ERINI platform (Grasse, France) using a Waters UPLC coupled with a Waters (Milford, MA, USA) Xevo G2 QTOF spectrometer.

Enantioselective SFC was performed on a Jasco (Tokyo, Japan) Extrema apparatus equipped with Daicel (Osaka, Japan) ChiralPak IA column coupled with a dual wavelength 190 to 600 nm UV-4070/75 detector. Pressure: 150 bars; flow: 4 mL/min; MeOH: 15%. Wavelength: 245 nm.

Polarimetry was performed on an Anton Paar (Graz, Austria) MCP150 polarimeter at 20 °C in MeOH/H_2_O 10:6 *v/v*. Wavelength: 589 nm.

Materials. Dimethyl formamide (DMF), tetrahydrofuran (THF), methanol (MeOH), Ethanol (EtOH), cyclohexane (CHX) were purchased from Sigma-Aldrich (Saint-Louis, MO, USA) and dried and/or distilled according to conventional procedures. Orcinol, POCl_3_, MOMCl, NaH, n-BuLi, AcCl, Na_2_CO_3_, NaHCO_3_, H_2_O_2_ (30% *w*/*w* in water) and HRP were purchased from Sigma-Aldrich and used as received.

*1,3-di(methoxymethoxy)-5-methylbenzene*: Orcinol (0.8 g; 6.5 mmol) was dissolved in freshly dried and distilled DMF (40 mL). To this solution was added NaH (0.58 g as a 60% dispersion in mineral oil, 14.5 mmol). The mixture was stirred under a nitrogen atmosphere at 0 °C in a round-bottomed flask equipped with a refrigerant and a bubbler allowing to monitor H_2_ evolution. After 15 min, MOM-Cl (1.10 mL; 14.5 mmol) was added and the mixture stirred during pendant 18 h. Water was then carefully added (30 mL) and the resulting mixture extracted with Et_2_O (5 × 30 mL). Organic layers were then pooled and washed with a 2 M aqueous NaOH solution (3 × 20 mL) and brine (20 mL). After drying over MgSO_4_, filtration and solvent removal, a yellow oil was obtained, which was submitted to column chromatography over silica gel (petroleum ether/EtOAc 9:1) to yield the MOM-protected orcinol as a colourless liquid (1.13 g, 95%). Rf 0.7 (petroleum ether/EtOAc 8:2). ^1^H NMR (CDCl_3_, 200 MHz): δ ppm 6.6–6.5 (m, 3H, ArH), 5.15 (s, 4H), 3.48 (s, 6H) 2.32 (s, 3H). ^13^C NMR: (CDCl_3_, 50 MHz) δ ppm 158.2 (C), 140.3 (C), 110.4 (CH), 102.1 (CH), 94.4 (CH), 55.9 (OCH_3_), 21.7 (CH_3_); MS (EI) *m/z:* 212(8), 182(1), 152(3), 136(2), 123(1), 108(2), 91(1), 77(2), 45(100).

*1,3-di(methoxymethoxy)-4-methylbenzaldehyde*: 1,3-di(methoxymethoxy)-5-methylbenzene (0.95 g; 4.5 mmol) was dissolved in freshly distilled THF (50 mL) and stirred under a nitrogen atmosphere at 0 °C while *n*-butyllithium was added dropwise (3.4 mL as a 1.6 M solution in hexane, 5.4 mmol). The mixture was stirred during 1.5 h while let warm to room temperature and the reaction was quenched with DMF (0.7 mL, 9 mmol). The resulting mixture was washed with water (50 mL) and extracted with Et_2_O (4 × 30 mL). Organic layers were pooled and washed with water (40 mL) and brine (40 mL), dried over MgSO_4_, filtrated and concentrated in vacuo. The resulting yellow oil was submitted to column chromatography over silica gel (petroleum ether/EtOAc 95:5) to yield a yellow solid (0.81 g, 75%). ^1^H NMR (CDCl_3_, 200 MHz): δ ppm 10.40 (s, 1H, CHO), 6.58 (s, 2H, ArH), 5.17 (s, 4H), 3.42 (s, 6H) 2.26 (s, 3H); ^13^C NMR: (CDCl_3_, 50 MHz) δ ppm 188.7 (CHO), 159.4 (C), 147.3 (C), 113.7 (CH), 109.3 (CH), 94.6 (CH), 56.3 (OCH_3_), 22.5 (CH_3_); MS *m*/*z:* 240(2), 209(2), 195(3), 179(4), 178(10), 165(3), 164(4), 136(2), 77(2), 46(3), 45(100).

*Atranol*: 1,3-di(methoxymethoxy)-4-methylbenzaldehyde (0.315 g, 1.3 mmol) was dissolved in 30 mL of MeOH at room temperature under a nitrogen atmosphere. Acetyl chloride was then added dropwise (60 µl, 0.9 mmol). The mixture was stirred for 20 h and concentrated in vacuo. An aqueous HCl solution was then added (30 mL at 0.1 M) and the resulting mixture extracted with EtOAc (3 × 150 mL). Organic layers were pooled and dried over MgSO_4_, filtrated and concentrated in vacuo. The resulting light-yellow oil was submitted to column chromatography over silica gel (petroleum ether/EtOAc 8:2), and atranol 1 was obtained as a light yellow oil (0.164 g, 90%). ^1^H NMR (Acetone-d_6_, 200 MHz): δ ppm 10.71 (s, 2H, OH), 10.26 (s, 1H, CHO), 6.25 (s, 2H, ArH), 2.23 (s, 3H). ^13^C NMR (Acetone-d_6_, 50 MHz): δ ppm 194.2 (CHO), 163.1 (C), 151.6 (C), 109.3 (C), 108.4 (CH), 22.4 (CH_3_). MS *m/z*: 152(84), 151(100), 134 (6), 123(4), 106 (16), 95(9), 77(14), 69 (6), 67(11), 55(12).

*5-Methylpyrogallol***1**: Atranol (0.152 g, 1 mmol) and sodium percarbonate (0.236 g, 1.5 mmol) were dissolved in THF/water 3:7 mixture (5 mL). The reaction mixture is then stirred at room temperature for 2 h. After completion of the reaction, aqueous 0.1 M HCl solution was added (5 mL) and the mixture extracted with EtOAc (2 × 10 mL). Organic layers were pooled and dried over MgSO_4_, filtrated and concentrated in vacuo. An orange solid was obtained (0.119 g, 85%). ^1^H NMR (Acetone-d_6_, 200 MHz): δ ppm 7.64 (s, 2H, OH), 7.02 (s, 1H, OH), 6.20 (s, 2H, ArH), 2.10 (s, 3H). ^13^C NMR (Acetone-d_6_, 50 MHz): δ ppm 146.3 (C), 131.0 (C), 129.2 (C), 108.5 (CH), 20.9 (CH_3_). MS *m*/*z*: 140(100), 139(34), 134 (6), 123(11), 122(19), 121(9), 94 (35), 77(4), 66(37), 65(21), 55(6), 53(17).

*Dimer***2**: 5-methylpyrogallol (200 mg, 1.43 mmol) was dissolved in a carbonate aqueous buffer at pH9 (20 mM, 65 mL), containing HRP (124 U/mg, 4 mg). The flask was covered with an aluminium foil and H_2_O_2_ (30% aqueous solution) was added at the controlled flow of 0.1 mL/h to reach a final amount of 2 equiv. The reaction mixture was stirred at room temperature for 6 h followed by the addition of an aqueous HCl solution (40 mL, 0.1 M) further extracted with EtOAc (3 × 70 mL). Evaporation of aqueous phase a white powder of dimer **2** and small amounts of salt (0.193 g, 88%). ^1^H NMR (D_2_O, 400 MHz): δ ppm 6.37 (t, 1H), 3.97 (d, 1H), 3.37 (s, H), 2.68 (s, 2H), 2.62 (s, H), 2.14 (s, 3H), 1.79 (s, 3H). ^13^C RMN: (D_2_O, 100 MHz) δ ppm 197.2 (C), 178.0 (C), 171.5 (C), 164.1 (C), 127.1 (CH), 88.7 (C), 87.1 (C), 85.7 (C), 77.7 (C), 60.1 (CH), 54.4 (CH), 52.9 (CH), 25.7 (CH_3_), 23.6 (CH_3_). MS (ESI): [M − H]^−^ ion was observed at *m*/*z* 309, [2M − H]^+^ at *m*/*z* 619, [2M − 2H + Na]^+^ at *m*/*z* 641. A (20 °C, 589 nm) = −0.002° ± 0.000.

*Acetylated dimer***2′**: To a solution of dimer **2** (0.100 g, 0.32 mmol) in freshly distilled dichloromethane (2 mL) were added Et_3_N (0.28 mL, 2.1 mmol) and Ac_2_O (0.2 mL, 2.1 mmol). The mixture was stirred overnight at room temperature under a nitrogen atmosphere. After concentration in vacuo, water was added (10 mL) and the resulting solution extracted with EtOAc (2 × 10 mL). The organic layers were pooled, dried over MgSO_4_, filtrated and concentrated in vacuo. A colourless oil was obtained (0.12 g, 90%). ^1^H NMR (CDCl_3_, 400 MHz): δ ppm 6.12 (s, 1H), 5.26 (s, 1H), 5.16 (s, 1H), 3.88 (s, 1H), 3.86 (s, 1H), 3.62 (s, 1H), 2.23 (s, 3H), 2.10 (s, 3H), 2.05 (s, 3H), 1.67 (s, 3H). ^13^C NMR: (CDCl_3_, 100 MHz) δ ppm 170.9 (CO), 169.8 (CO), 169.7 (CO), 169.5 (CO), 169.1 (CO), 140.6 (C), 134.3 (C), 120.9 (C), 88.6(C), 87.3(C), 80.9 (C), 77.7 (C), 58.6 (CH), 55.5 (CH), 50.6 (CH), 23.2 (CH_3_), 20.9 (CH_3_), 20.7 (CH_3_), 20.7 (CH_3_), 20,5 (CH_3_). MS *m*/*z*: 418(1), 376(20), 334(7), 290(2), 273(3), 266(1), 246(2), 231(3), 214(6), 182(23), 175(3), 162(6), 141(28), 140(100), 91(1), 77(2), 69(2), 43(100). HRMS: 419.0950, calculated for [M.H]^+^ C_20_H_19_O_10_ 419.0978, ∆ = −6.7 ppm. 377.0859, calculated for [M(-CH_3_CO + H).H]^+^ C_18_H_17_O_9_ 377.0873, ∆ = −3.7 ppm. 335.0754, calculated for [M(-2CH_3_CO + 2H).H]^+^ C_16_H_15_O_8_ 335.0767, ∆ = −3.9 ppm.

## 4. Conclusions

In summary, an unusual complex fused polycyclic new skeleton featuring a 2-oxatetracyclo [6.3.1.0^1,6^.0^4,12^] dodecan-3-one was obtained in one-step from 5-methylpyrogallol submitted to HRP-catalysed oxidation in the presence of H_2_O_2_. The impressive control of the regio- and stereoselectivity of all the putative steps of this transformation reflects the unique capacity of enzymes in organic chemistry and their potential for building outstanding chemical skeletons by triggering complex cascades proceeding selectively either under an enzyme-controlled regime or substrate-controlled regime. In our case, the enzymatic reaction would catalyse the formation of highly activated species further undergoing spontaneous reactions, a scenario that could also be considered in biosynthetic studies [17,18]. The production of such a chemical complexity from a simple and close derivative of natural gallic acid could also later unravel structural analogues from their natural origin.

## Figures and Tables

**Table 1:** Testing of HRP-catalysed oxidation of 5-methylpyrogallol **1**.

Entry	H_2_O_2_	HRP	Outcome
1	-	-	Recovery of SM 91%
2	-	1% (*w*/*w*)	Recovery of SM 95%
3	2 equiv.	-	Recovery of SM 34%
4	2 equiv.	1% (*w*/*w*)	Dimer 2, 89% ^a^
5	2 equiv.	2% (*w*/*w*)	Dimer 2, 86% ^a^
6	2 equiv.	5% (*w*/*w*)	Dimer 2, 88% ^a^
7	2 equiv.	10% (*w*/*w*)	Dimer 2, 91% ^a^
8	3 equiv.	1% (*w*/*w*)	Dimer 2, 81% ^a^
9	4 equiv.	1% (*w*/*w*)	Dimer 2, 92% ^a^
10	5 equiv.	1% (*w*/*w*)	Dimer 2,87% ^a^

^a^ Obtained after evaporation of the aqueous phase.

**Table 2:**
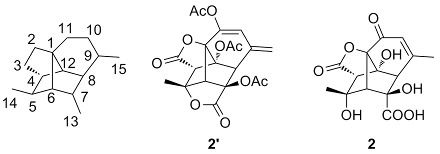
^1^H and ^13^C-NMR chemical shifts of **2′**.

2′ ^a^					
Entry	Position ^b^	^1^H	^13^C	Description	HMBC
1	-	-	170.9	CH_3_O-**C=O**	
2	-	-	169.8	CH_3_O-**C=O**	
3	-	-	169.7	CH_3_O-**C=O**	
4	11	-	140.6	>C=	
5	3	-	169.1	O-C=O	
6	13	-	169.5	O-C=O	
7	9	-	134.3	>C=	
8	10	6.12	118.5	=CH-	C-11, C-1, C-8, C-9, C-15
9	15	5.26, 5.16	120.9	0	C-8, C-9, C-10
10	1	-	77.7	>C<	
11	12	-	88.6	>C<	
12	5	-	80.9	>C<	
13	7	-	87.3	>C<	
14	4	3.62	58.6	CH	C-6, C-5, C-1, C-12
15	6	3.88	55.5	CH	C-13, C-7, C-5, C-4, C-14, C-12
16	8	3.86	50.6	CH	C-12, C-1, C-7, C-13
17	14	1.67	23.2	CH_3_	C-6, C-5, C-4
18	-	2.05	20.9	OCH_3_	
19	-	2.1	20.7	OCH_3_	
20	-	2.23	20.5	OCH_3_	

^a^ CDCl_3_, 400 and 100 MHz, respectively, δ ppm. HMBC = Heteronuclear Multiple Bond Correlation. ^b^ The numbering system is that of the parent polycyclic system, the tetracyclo [6.3.1.0^1,6^.0^4,12^] dodecane core. For an alternate numbering system, see supplementary materials (SM).

**Table 3:** ^1^H and ^13^C-NMR chemical shifts of **2**.

2 ^a^				
Entry	Position ^b^	^1^H	^13^C	Description
1	11	-	197.2	>C=O
2	13	-	178.0	HO-C=O
3	3	-	171.5	O-C=O
4	9	-	164.1	>C=
5	10	6.45	127.1	=CH-
7	1	-	88.7	>C<
8	12	-	87.1	>C<
9	5	-	85.7	>C<
10	7	-	77.7	>C<
11	4	2.67	60.1	CH
12	6	3.57	54.4	CH
13	8	3.95	52.9	CH
14	15	2.18	25.7	CH_3_
15	14	1.83	23.6	CH_3_

^a^ D_2_O, 400 and 100 MHz, respectively, δ ppm. ^b^ The numbering system is that of the parent polycyclic system, the tetracyclo [6.3.1.0^1,6^.0^4,12^] dodecane core.

## Supplementary Materials

The following are available online. Detailed experimental procedures and NMR spectra of products and intermediates.
