# Supplementary material for: Unusual Polycyclic Fused Product by Oxidative Enzymatic Dimerisation of 5-methylpyrogallol Catalysed by Horseradish Peroxidase/H2O2
Source: Molecules. 2018 Oct 12;23(10):2619. doi: 10.3390/molecules23102619 (PMC6222440; doi:10.3390/molecules23102619)
Supplement: Supplementary file 1 [file molecules-23-02619-s001.pdf]

Unusual Polycyclic Fused Product by Oxidative Enzymatic Dimerisation of 5-Methylpyrogallol catalyzed by Horseradish Peroxidase/H_2O_2

Hélène Bouges,[†] Kevin Calabro,[‡] Olivier P. Thomas,[‡] and Sylvain Antoniotti*[†]

[†] Université Cote d'Azur.

[‡] National University of Ireland Galway. Supporting Information

Supporting information: Table of contents

1. Equipment…… 2
2. Synthesis of 5-methylpyrogallol from orcinol………………………………………………………… 3
Figure S 1. ^{1}H-NMR spectrum of 1,3-di(methoxymethoxy)-5-methylbenzene……………………………. 3
Figure S 2. ^{13}C-NMR spectrum of 1,3-di(methoxymethoxy)-5-methylbenzene……………………………. 4
Figure S 3. ^{1}H-NMR spectrum of 1,3-di(methoxymethoxy)-4-methylbenzaldehyde…………………… 5
Figure S 4. ^{13}C-NMR spectrum of 1,3-di(methoxymethoxy)-4-methylbenzaldehyde…………………… 5
Figure S 5. ^{1}H-NMR spectrum of atranol………………………………………………………………………. 6
Figure S 6. ^{13}C-NMR spectrum of atranol……………………………………………………………………… 7
Figure S 7. ^{1}H-NMR spectrum of isoatranol…………………………………………………………………… 8
Figure S 8. ^{13}C-NMR spectrum of isoatranol…………………………………………………………………. 8
Figure S 9. ^{1}H-NMR of 5-methylpyrogallol **1**………………………………………………………………….. 9
Figure S 10. ^{13}C-NMR of 5-methylpyrogallol **1**……………………………………………………………… 10
3. Dimerisation of 5-methylpyrogallol…………………………………………………………………… 11
Figure S 11. HPLC-MS analysis of **2**……………………………………………………………………………… 11
Figure S 12. Enantioselective-SFC analysis of dimer **2**……………………………………………………… 12
Figure S 13. ^{1}H-NMR spectrum of **2**…………………………………………………………………………… 13
Figure S 14. ^{13}C-NMR spectrum of **2**………………………………………………………………………… 14
Figure S 15. HMQC spectrum of **2**……………………………………………………………………………… 15
Figure S 16. HMBC spectrum of **2**……………………………………………………………………………… 16
Figure S 17. HMRS spectrum of **2'**……………………………………………………………………………… 17
Figure S 18. ^{1}H-NMR spectrum of **2'**…………………………………………………………………………. 18
Figure S 19. ^{13}C-NMR spectrum of **2'**……………………………………………………………………… 19
Figure S 20. HMQC spectrum of **2'**……………………………………………………………………………. 20
Figure S 21. HMBC spectrum of **2'**……………………………………………………………………………. 21
Figure S 22. COSY of **2'**…………………………………………………………………………………………. 22
Figure S 23. HMQC correlation for **2'**…………………………………………………………………………. 23
Table S 1. ^{1}H and ^{13}C-NMR chemical shifts of **2'** with alternative numbering system………………… 23

1. Equipment

^1H NMR and ^{13}C NMR spectra were recorded on a BRUCKER AC (200 and 400 MHz). ^1H NMR spectra are reported as follows: chemical shifts in ppm (δ) relative to the chemical shift of TMS at 0 ppm, integration, multiplicities (s = singlet, d = doublet, t = triplet, q = quartet, m = multiplet and br = broad), and coupling constants (Hz). ^{13}C NMR spectra chemical shifts are reported in ppm (δ) relative to $CDCl_3$ at 77.16 ppm. Identity was assessed by comparison with data of authentic samples or literature data for **1** and its synthetic intermediates.

Column chromatography was carried out on silica gel (spherical 15-30 µm, neutral, 63–200 µm, Geduran Si 60, Merck KGaA).

GC-TCD analyses were carried out using a Shimadzu QP2010 plus gas chromatograph, under the following operation conditions: vector gas, He; injector temperature, 250 °C; detector temperature, 210 °C at 60 mA; split ratio, 1/20; total flow, 22.5 ml min−1; Phenomenex Zebron ZB5MS column, polydimethylsiloxane (10 m, inside diameter 0.10 mm, film thickness 0.10 µm); temperature program, 80–200 °C at 10 °C min−1 and 200 °C for 8 min.

GC/MS analyses were performed by using a Shimadzu QP2010 gas chromatograph (conditions: carrier gas, He; injector and detector temperatures, 250 °C; injected volume, 0.5 µL; split ratio, 1/100; pressure, 180 kPa; SLB-5ms capillary column (thickness: 0.25 mm, length: 30 m, inside diameter: 0.25 mm); temperature program, 60–315 °C at 10 °C min−1, and 10 min at 315 °C) coupled to a mass selective detector. Mass spectra were obtained by electron ionisation at 70 eV, m/z 35–400, source temperature 250 °C; only the most abundant ions are given.

High resolution mass spectrometry (HRMS) was performed at ERINI platform (Grasse, FRANCE) using a Waters UPLC coupled with a Waters Xevo G2 QTOF spectrometer.

Enantioselective SFC was performed on a Jasco Extrema apparatus equipped with Daicel ChiralPak IA column coupled with a dual wavelength 190 to 600 nm UV-4070/75 detector. Pressure: 150 bars; flow: 4mL/min; MeOH: 15%. Wavelength: 245nm.

Polarimetry was performed on an Anton Paar MCP150 polarimeter at 20 °C in MeOH/H_2O 10:6 v.v. Wavelength: 589 nm.

Materials. Dimethyl formamide (DMF), tetrahydrofuran (THF), methanol (MeOH), ethanol (EtOH), cyclohexane (CHX) were purchased from Sigma-Aldrich and dried and/or distilled according to conventional procedures. Orcinol, $POCl_3$, MOMCl, NaH, n-BuLi, AcCl, Na_2CO_3, $NaHCO_3$, H_2O_2 (30% w/w in water) and HRP were purchased from Sigma-Aldrich and used as received.

2. Synthesis of 5-methylpyrogallol from orcinol

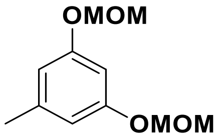

1,3-di(methoxymethoxy)-5-methylbenzene: Orcinol (0.8 g; 6.5 mmol) was dissolved in freshly dried and distilled DMF (40 mL). To this solution was added NaH (0.58 g as a 60% dispersion in mineral oil, 14.5 mmol). The mixture was stirred under a nitrogen atmosphere at 0 °C in a round-bottomed flask equipped with a refrigerant and a bubbler allowing to monitor H_2 evolution. After 15 min, MOM-Cl (1.10 mL; 14.5 mmol) was added and the mixture stirred during pendant 18 h. Water was then carefully added (30 mL) and the resulting mixture extracted with Et_2O (5x30 mL). Organic layers were then pooled and washed with a 2M aqueous NaOH solution (3x20 mL) and brine (20 mL). After drying over $MgSO_4$, filtration and solvent removal, a yellow oil was obtained, which was submitted to column chromatography over silica gel (petroleum ether/EtOAc 9:1) to yield the MOM-protected orcinol as a colorless liquid (1.13 g, 95%). Rf 0.7 (petroleum ether/EtOAc 8:2). **^{1}H NMR** ($CDCl_3$, 200 MHz): δ ppm 6.6-6.5 (m, 3H, ArH), 5.15 (s, 4H), 3.48 (s, 6H) 2.32 (s, 3H). **^{13}C NMR** ($CDCl_3$, 50 MHz): δ ppm 158.2 (C), 140.3 (C), 110.4 (CH), 102.1 (CH), 94.4 (CH), 55.9 (OCH_3), 21.7 (CH_3). **MS (EI)** ***m/z:*** 212 (8), 182(1), 152(3), 136(2), 123(1), 108(2), 91(1), 77(2), 45 (100).

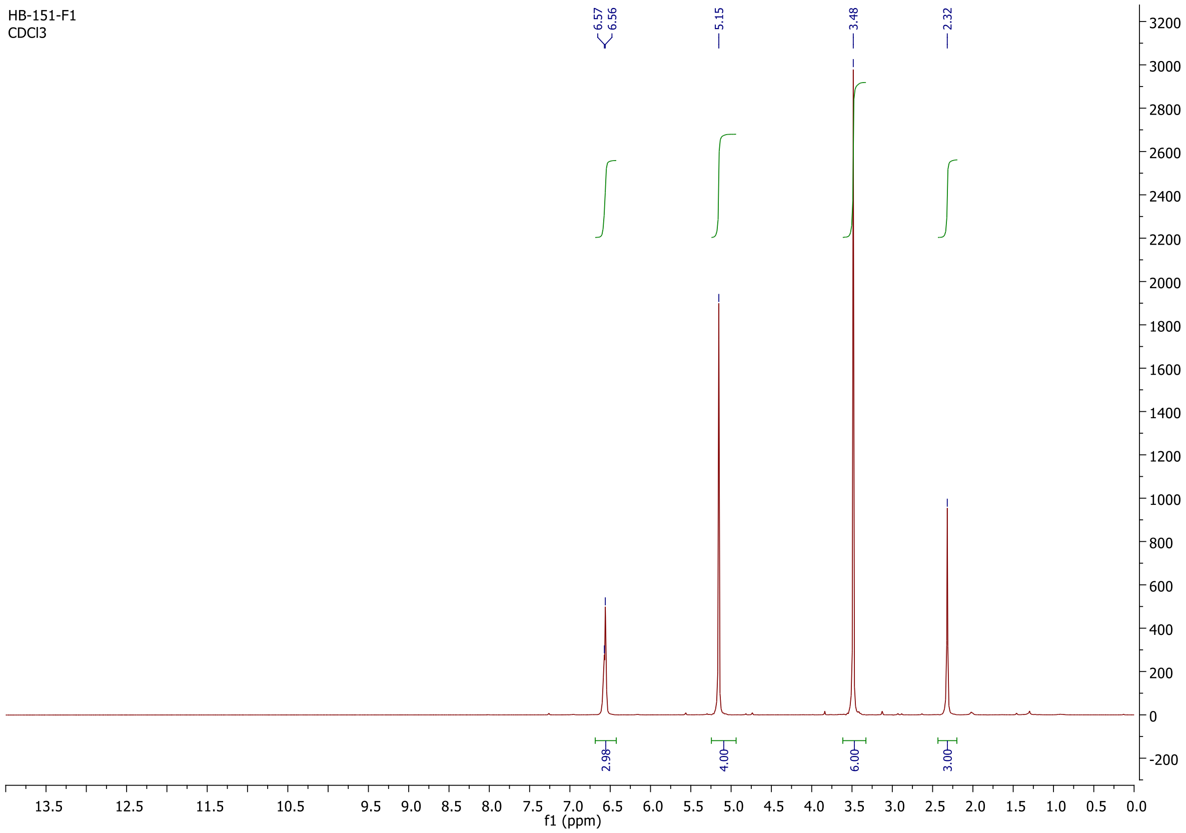

Figure S 1. ^{1}H-NMR spectrum of 1,3-di(methoxymethoxy)-5-methylbenzene.

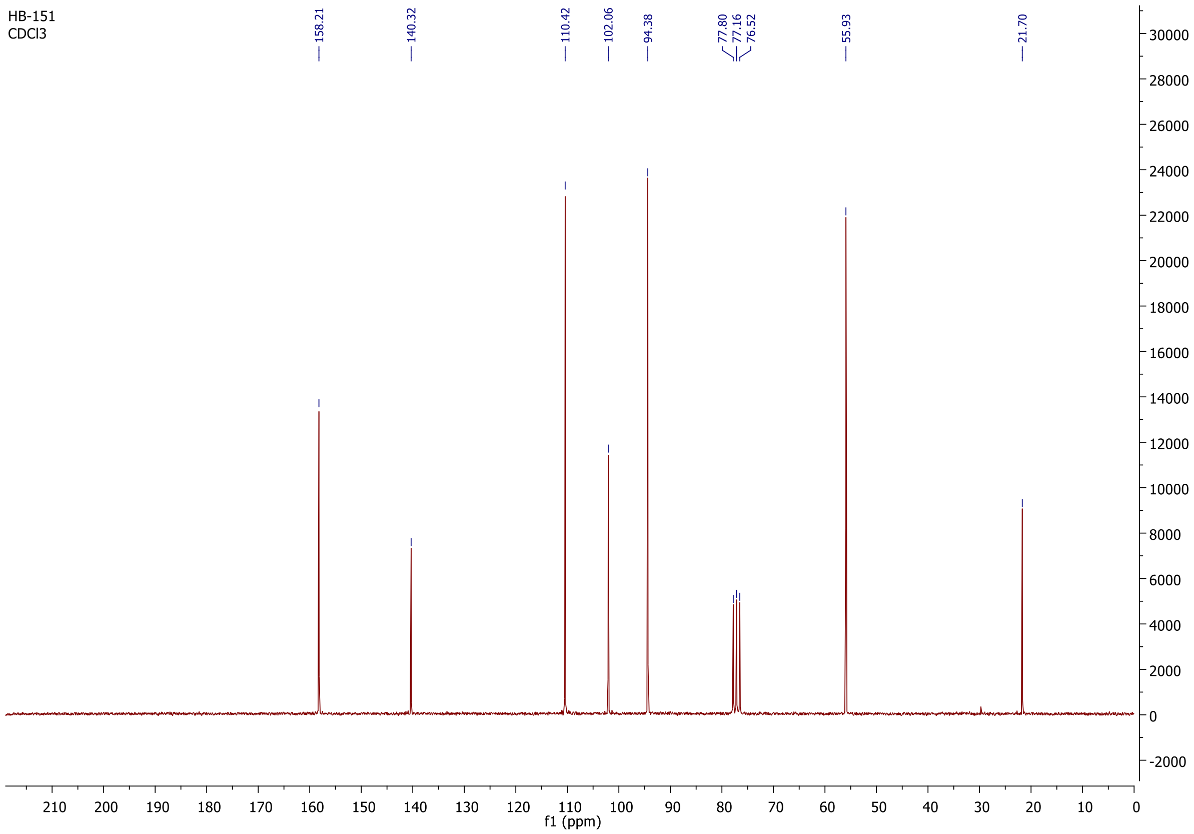

Figure S 2. ^{13}C-NMR spectrum of 1,3-di(methoxymethoxy)-5-methylbenzene.

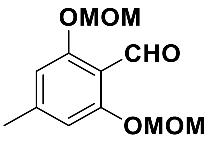

1,3-di(methoxymethoxy)-4-methylbenzaldehyde: 1,3-di(methoxymethoxy)-5-methylbenzene (0.95 g; 4.5 mmol) was dissolved in freshly distilled THF (50 mL), and stirred under a nitrogen atmosphere at 0°C while *n*-butyllithium was added dropwise (3.4 mL as a 1.6 M solution in hexane, 5.4 mmol). The mixture was stirred during 1.5 h while let warm to room temperature and the reaction was quenched with DMF (0.7 mL, 9 mmol). The resulting mixture was washed with water (50 mL) and extracted with Et_2O (4 x 30 mL). Organic layers were pooled and washed with water (40 mL) and brine (40 mL), dried over $MgSO_4$, filtrated and concentrated *in vacuo*. The resulting yellow oil was submitted to column chromatography over silica gel (petroleum ether/EtOAc 95 :5) to yield a yellow solid (0.81 g, 75%). **^{1}H NMR** ($CDCl_3$, 200 MHz): δ ppm 10.40 (s, 1H, CHO), 6.58 (s, 2H, ArH), 5.17 (s, 4H), 3.42 (s, 6H) 2.26 (s, 3H). **^{13}C NMR** ($CDCl_3$, 50 MHz): δ ppm 188.7 (CHO), 159.4 (C), 147.3 (C), 113.7 (CH), 109.3 (CH), 94.6 (CH), 56.3 (OCH_3), 22.5 (CH_3). **MS (EI)** ***m/z**:* 240 (2), 209(2), 195 (3), 179(4), 178(10), 165(3), 164(4), 136(2), 77(2), 46(3), 45(100).

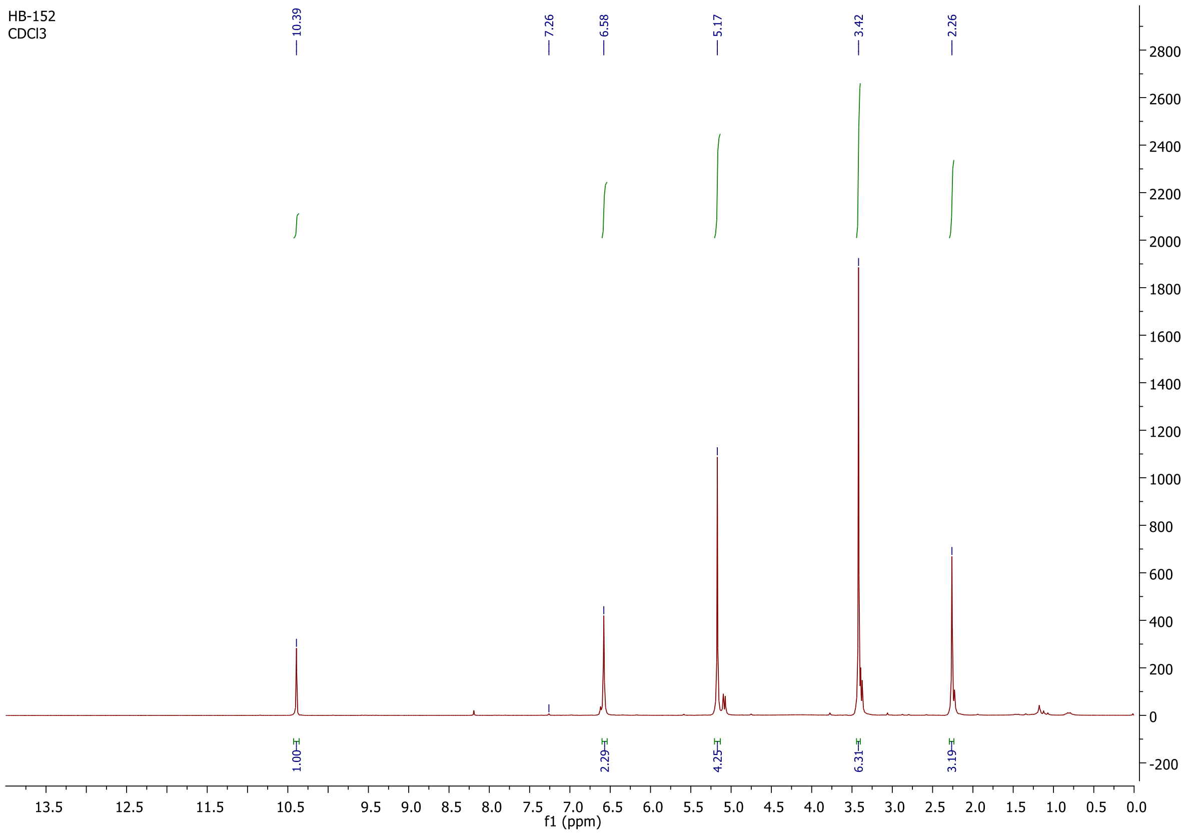

Figure S 3. ^{1}H-NMR spectrum of 1,3-di(methoxymethoxy)-4-methylbenzaldehyde.

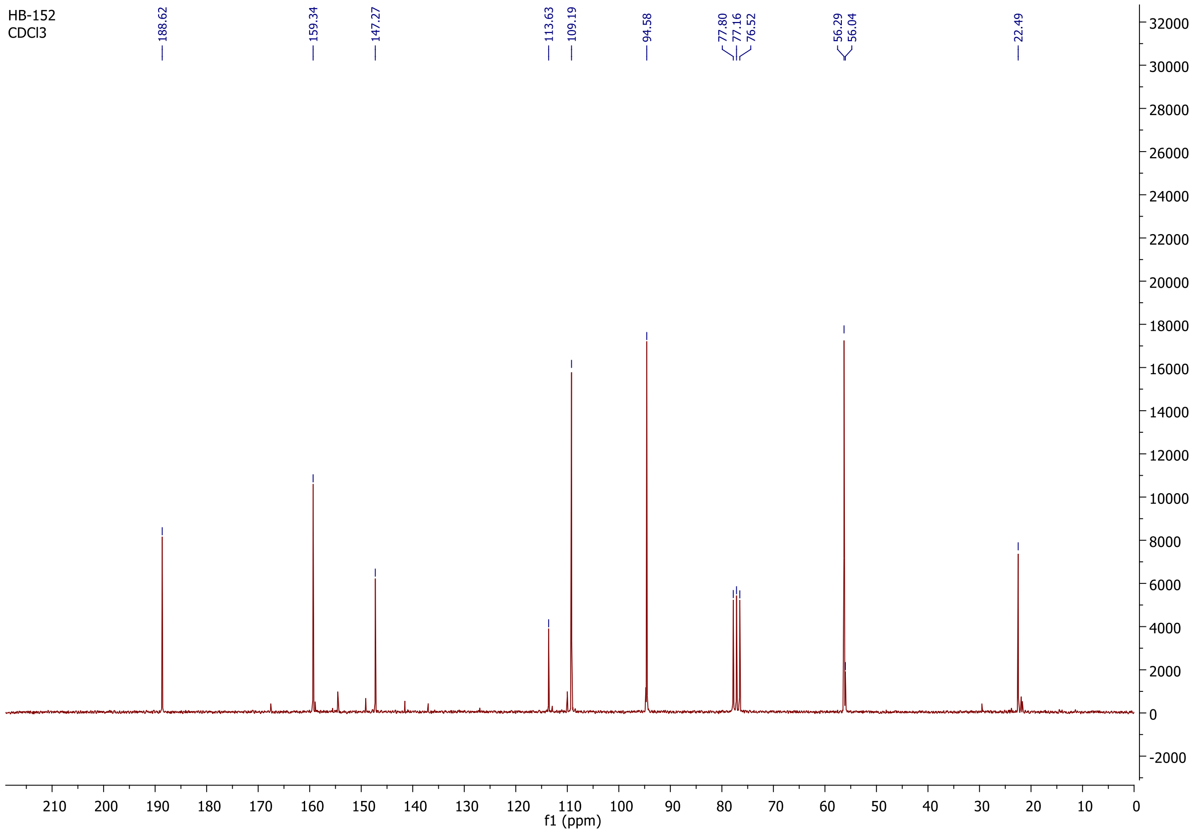

Figure S 4. ^{13}C-NMR spectrum of 1,3-di(methoxymethoxy)-4-methylbenzaldehyde.

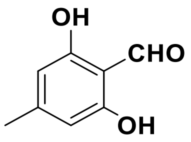

Atranol: 1,3-di(methoxymethoxy)-4-methylbenzaldehyde (0.315 g, 1.3 mmol) was dissolved in 30 mL of MeOH at room temperature under a nitrogen atmosphere. Acetyl chloride was then added dropwise (60 µl, 0.9 mmol). The mixture was stirred for 20 h and concentrated *in vacuo*. An aqueous HCl solution was then added (30 mL à 0,1 M) and the resulting mixture extracted with EtOAc (3 x 150 mL). Organic layers were pooled and dried over $MgSO_4$, filtrated and concentrated *in vacuo*. The resulting light yellow oil was submitted to column chromatography over silica gel (petroleum ether/EtOAc 8:2), and atranol **1** was obtained as a light yellow oil (0.164 g, 90%). **^{1}H NMR** (Acetone-d_6, 200 MHz): δ ppm 10.71 (s, 2H, OH), 10.26 (s, 1H, CHO), 6.25 (s, 2H, ArH), 2.23 (s, 3H). **^{13}C NMR** (Acetone-d_6, 50 MHz): δ ppm 194.2 (CHO), 163.1 (C), 151.6 (C), 109.3 (C), 108.4 (CH), 22.4 (CH_3). **MS (EI)** ***m/z****:* 152 (84), 151(100), 134 (6), 123(4), 106 (16), 95 (9), 77(14), 69 (6), 67(11), 55(12).

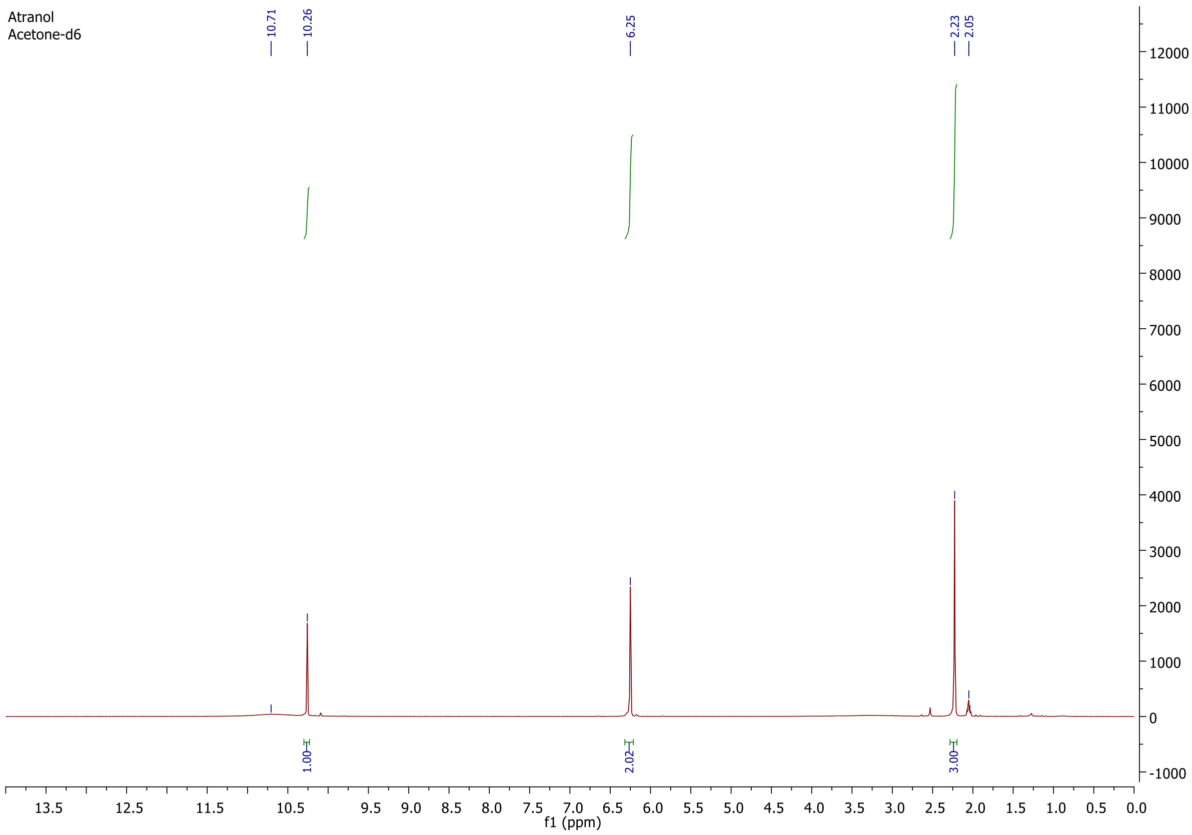

Figure S 5. ^{1}H-NMR spectrum of atranol.

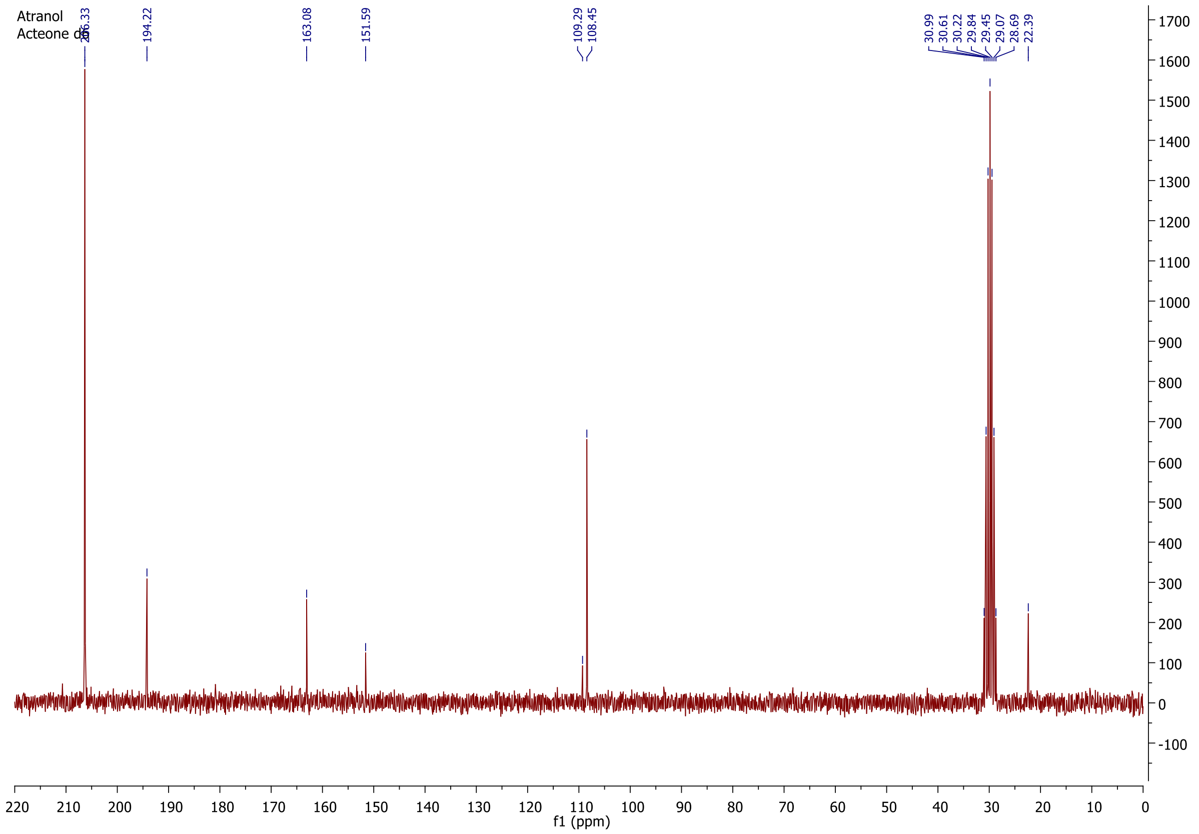

Figure S 6. ^{13}C-NMR spectrum of atranol.

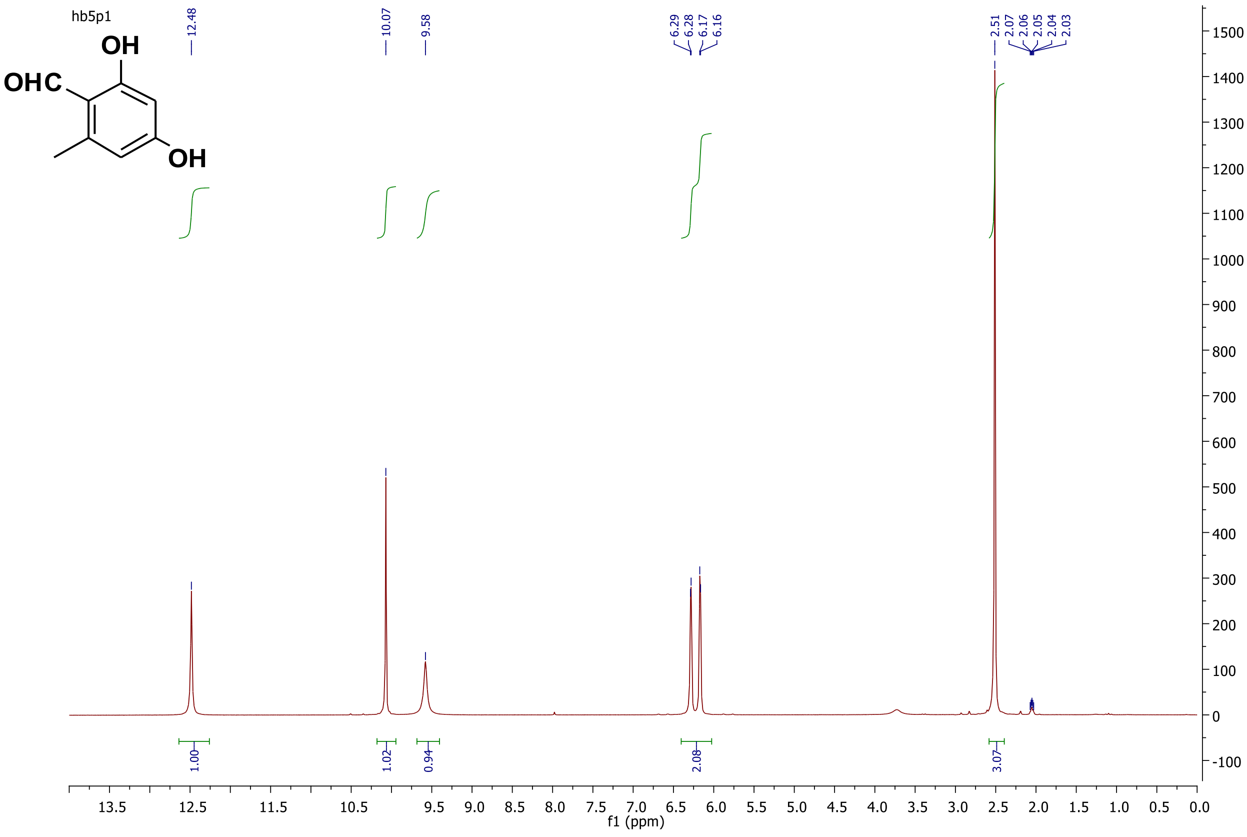

Figure S 7. ^{1}H-NMR spectrum of isoatranol.

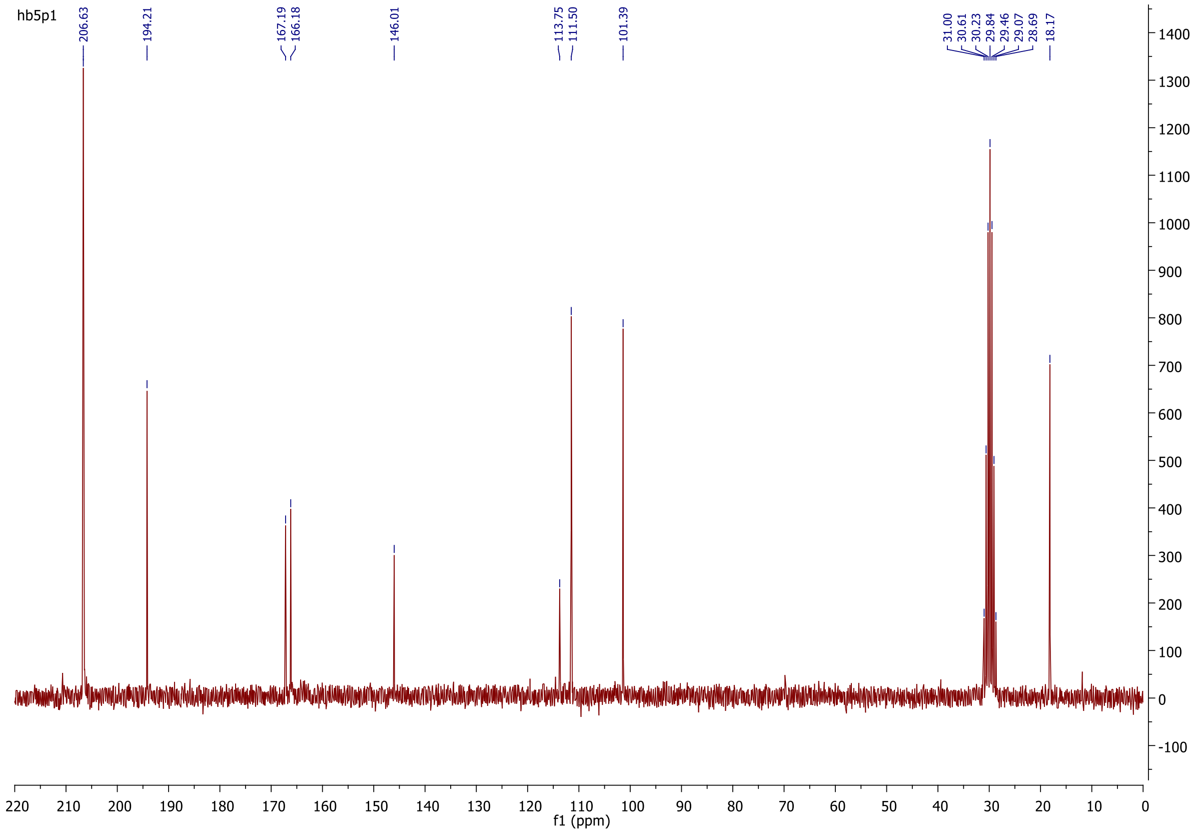

Figure S 8. ^{13}C-NMR spectrum of isoatranol.

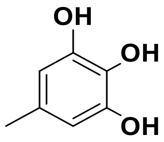

5-Methylpyrogallol 1: Atranol (0.152 g, 1 mmol) and sodium percarbonate (0.236 g, 1.5 mmol) were dissolved in THF/water 3:7 mixture (5 mL). The reaction mixture is then stirred at room temperature for 2 hours. After completion of the reaction, aqueous 0.1 M HCl solution was added (5 mL) and the mixture extracted with EtOAc (2x10 mL). Organic layers were pooled and dried over $MgSO_4$, filtrated and concentrated *in vacuo*. An orange solid was obtained (0.119 g, 85%). **^{1}H NMR** (Acetone-d_6, 200 MHz): δ ppm 7.64 (s, 2H, OH), 7.02 (s, 1H, OH), 6.20 (s, 2H, ArH), 2.10 (s, 3H). **^{13}C NMR** (Acetone-d_6, 50 MHz): δ ppm 146.3 (C), 131.0 (C), 129.2 (C), 108.5 (CH), 20.9 (CH_3). **MS (EI)** ***m/z:*** 140 (100), 139(34), 134 (6), 123(11), 122(19), 121 (9), 94 (35), 77(4), 66 (37), 65(21), 55(6), 53(17).

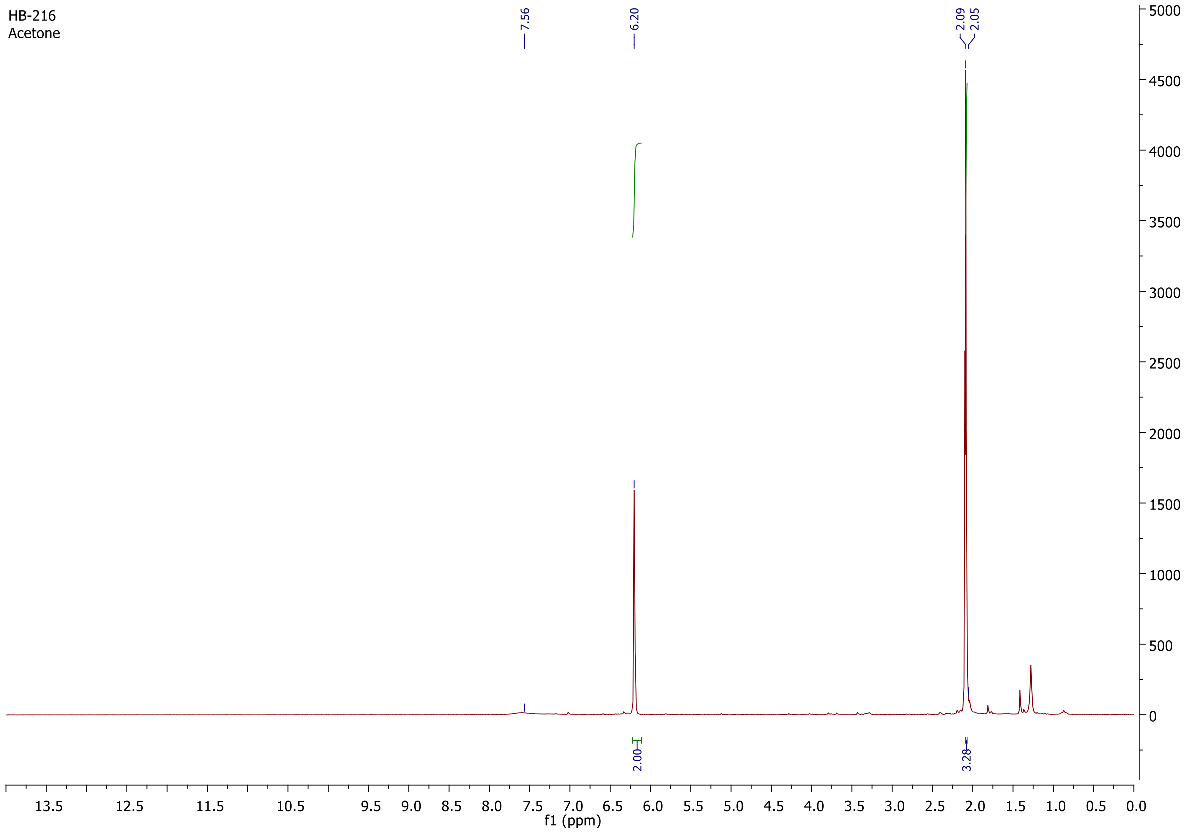

*Figure S 9. ^{1}H-NMR of 5-methylpyrogallol **1**.*

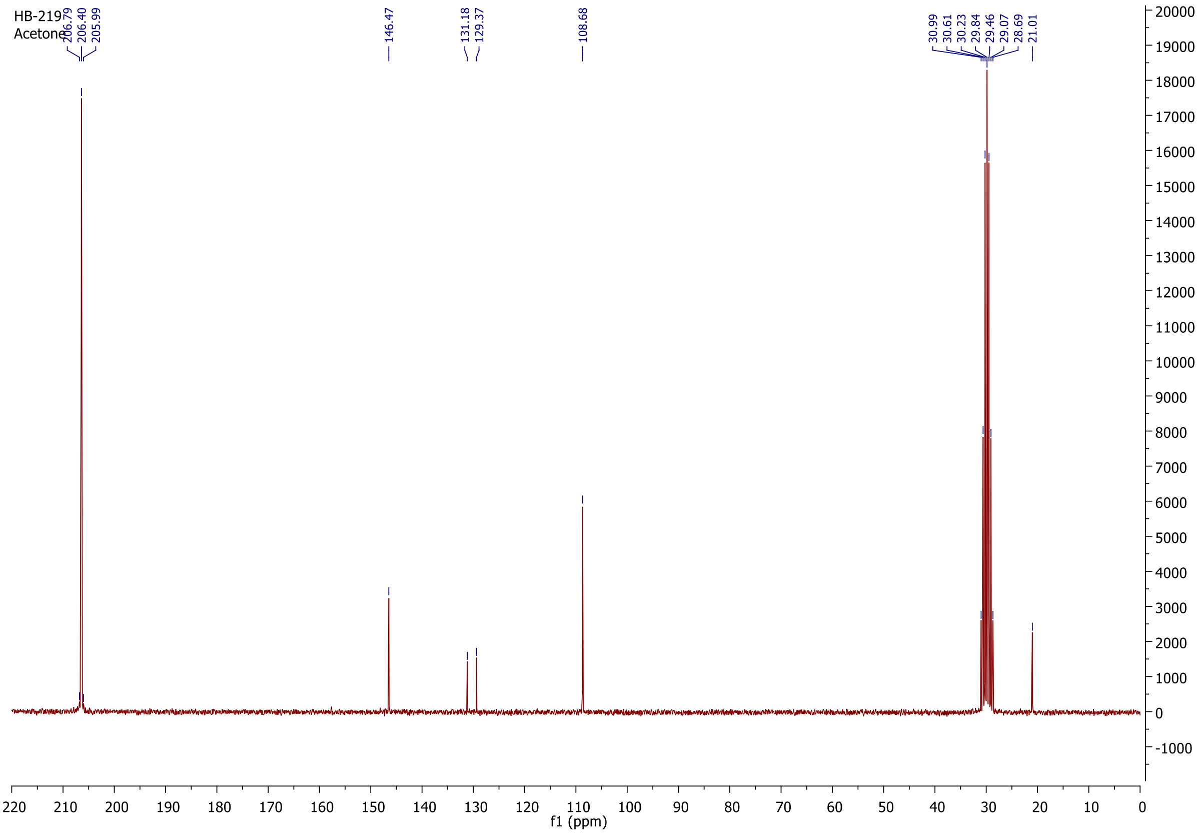

*Figure S 10. ^{13}C-NMR of 5-methylpyrogallol **1**.*

2. Dimerisation of 5-methylpyrogallol

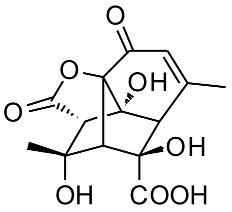

2

Dimer 2: 5-methylpyrogallol (200 mg, 1.43 mmol) was dissolved in a carbonate aqueous buffer at pH9 (20 mM, 65 mL), containing HRP (124 U/mg, 4 mg). The flask was covered with an aluminium foil and H_2O_2 (30% aqueous solution) was added at the controlled flow of 0.1 mL/h to reach a final amount of 2 equiv. The reaction mixture was stirred at room temperature for 6 hours followed by the addition of an aqueous HCl solution (40 mL, 0.1 M) further extracted with EtOAc (3x70 mL). Evaporation of aqueous phase led to a white powder (0.193 g, 88%).**^{1}H NMR** (D_2O, 400 MHz): δ ppm 6.37 (t, 1H), 3.97 (d, 1H), 3.37 (s, H), 2.68 (s, 2H), 2.62 (s, H), 2.14 (s, 3H), 1.79 (s, 3H). **^{13}C RMN** (D_2O, 100 MHz): δ ppm 197.2 (C), 178.0 (C), 171.5 (C), 164.1 (C), 127.1 (CH), 88.7 (C), 87.1 (C), 85.7 (C), 77.7 (C), 60.1 (CH), 54.4 (CH), 52.9 (CH), 25.7 (CH_3), 23.6 (CH_3). **MS** (ESI): $[M-H]^-$ ion was observed at *m/z* 309, $[2M-H]^+$ at *m/z* 619, $[2M-2H+Na]^+$ at *m/z* 641. α (20 °C, 589 nm)=-0,002° ±0.000 (triplicates).

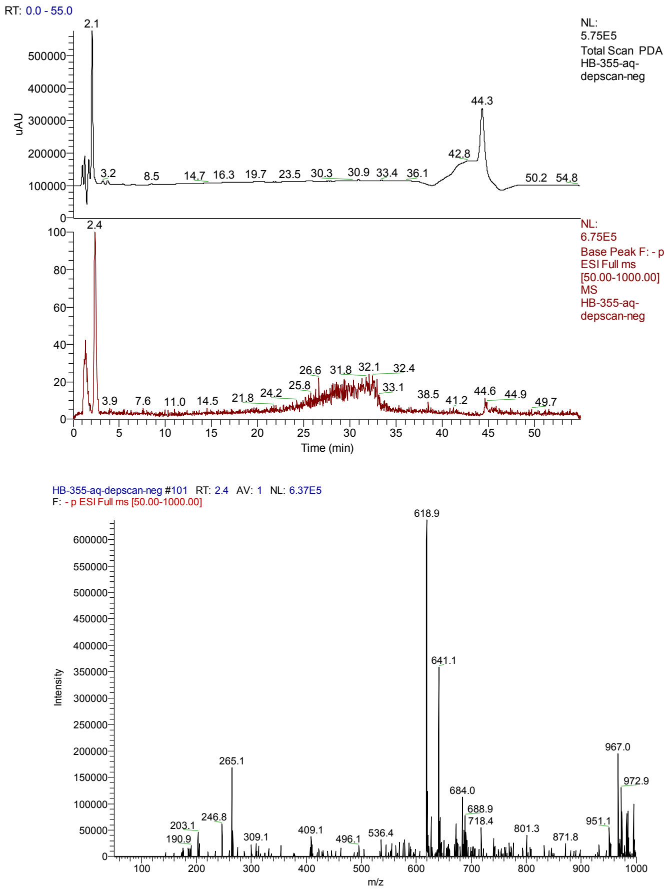

*Figure S 11. HPLC-MS of **2**.*

SA1-IA-150-4-15MeOH(245nm) SA1 27/07/2018 10:14:41

Chromatogram Information	
User Name	aurelien
Date Modified	27/07/2018 09:51:21
HPLC System Name	SFC-4000
Injection Date	27/07/2018 09:39:15
Volume	10,00 [µL]
Acquisition Time	25,0 [min]
Acquisition Sequence	SA1-IA-150-4-15MeOH(245nm)
Control Method	SFC-150-4-15MeOH (245nm)

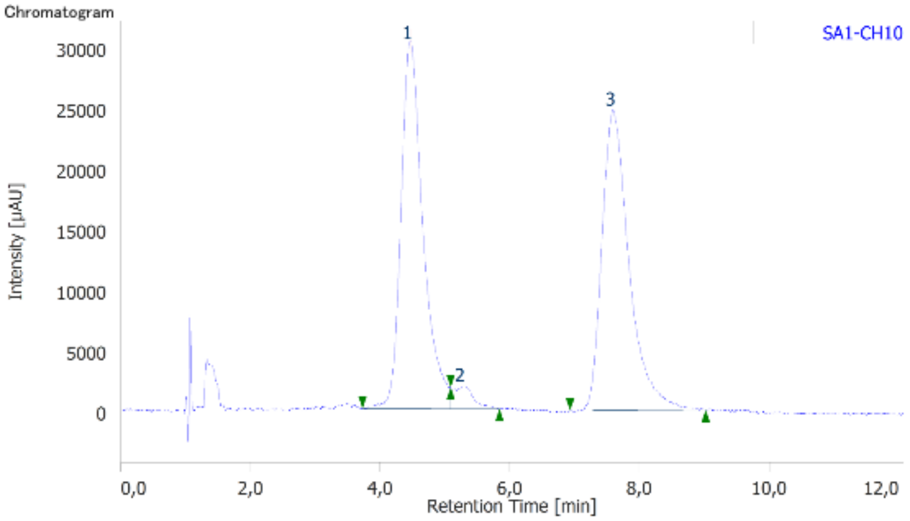

Peak Information

#	Peak Name	CH	tR [min]	Area [µV·sec]	Height [µV]	Area%	Height%	Quantity	NTP	Resolution	Symmetry Factor	Warning
1	Unknown	10	4,458	716302	30319	48,485	53,171	N/A	944	N/A	N/A	
2	Unknown	10	5,268	40138	1891	2,717	3,315	N/A	N/A	N/A	N/A	
3	Unknown	10	7,590	720932	24812	48,798	43,513	N/A	1736	N/A	1,518	

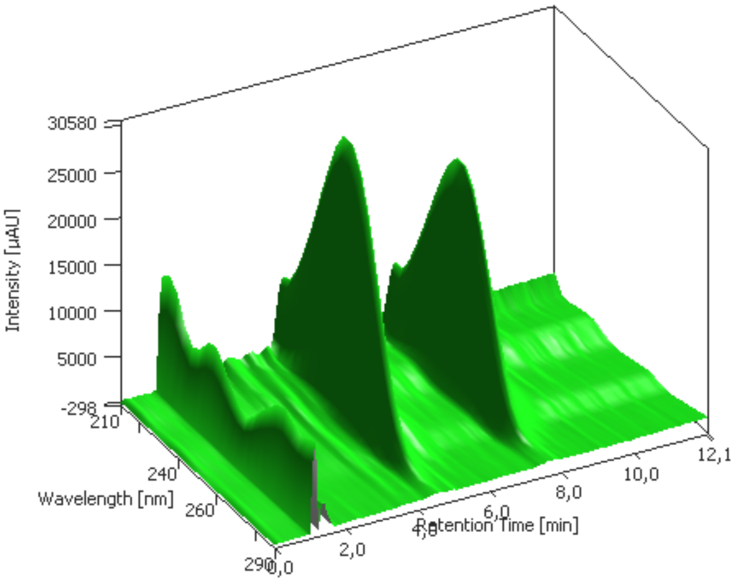

*Figure S 12. Enantioselective-SFC analysis of dimer **2**.*

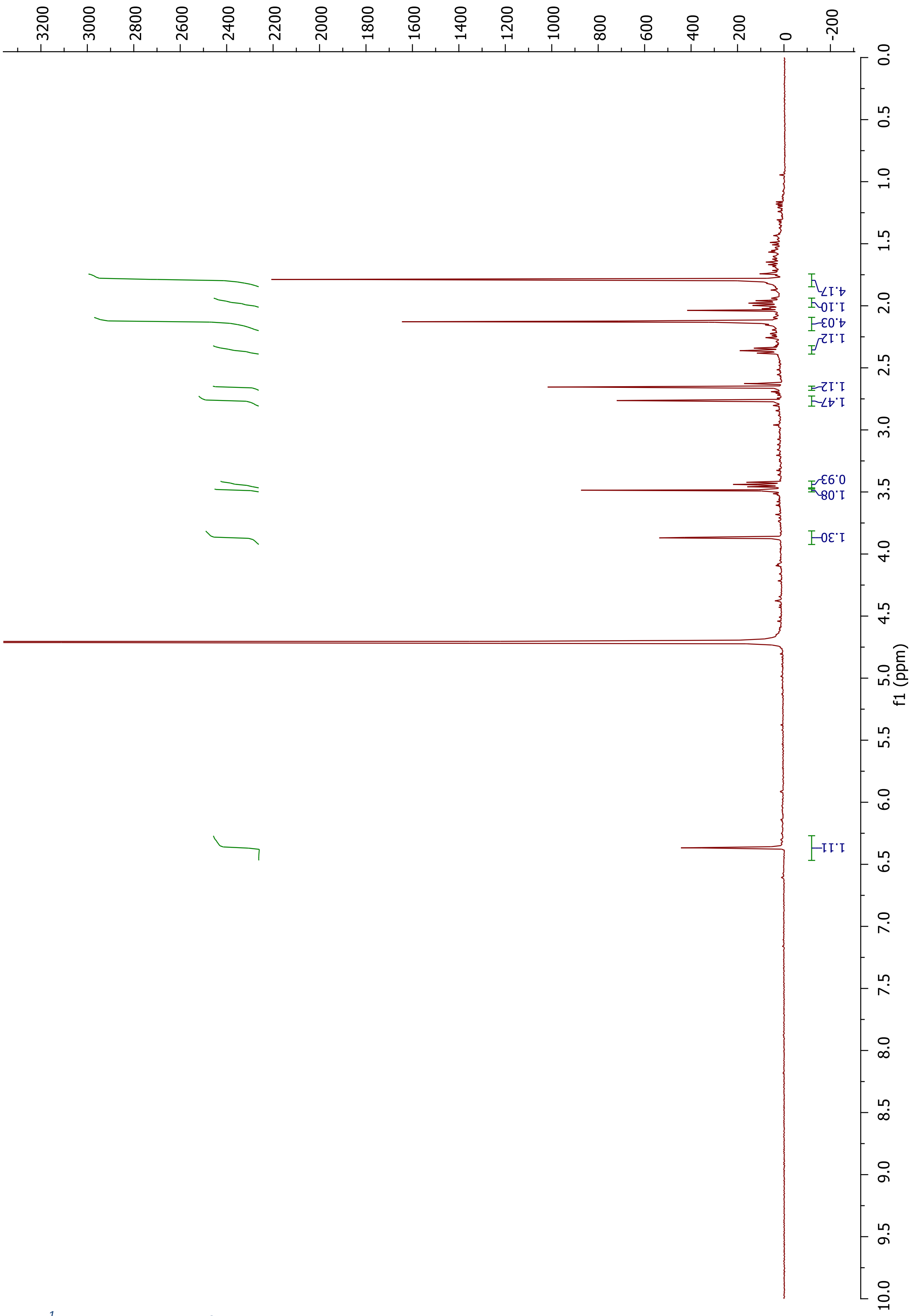

*Figure S 13. ^{1}H-NMR spectrum of **2**.*

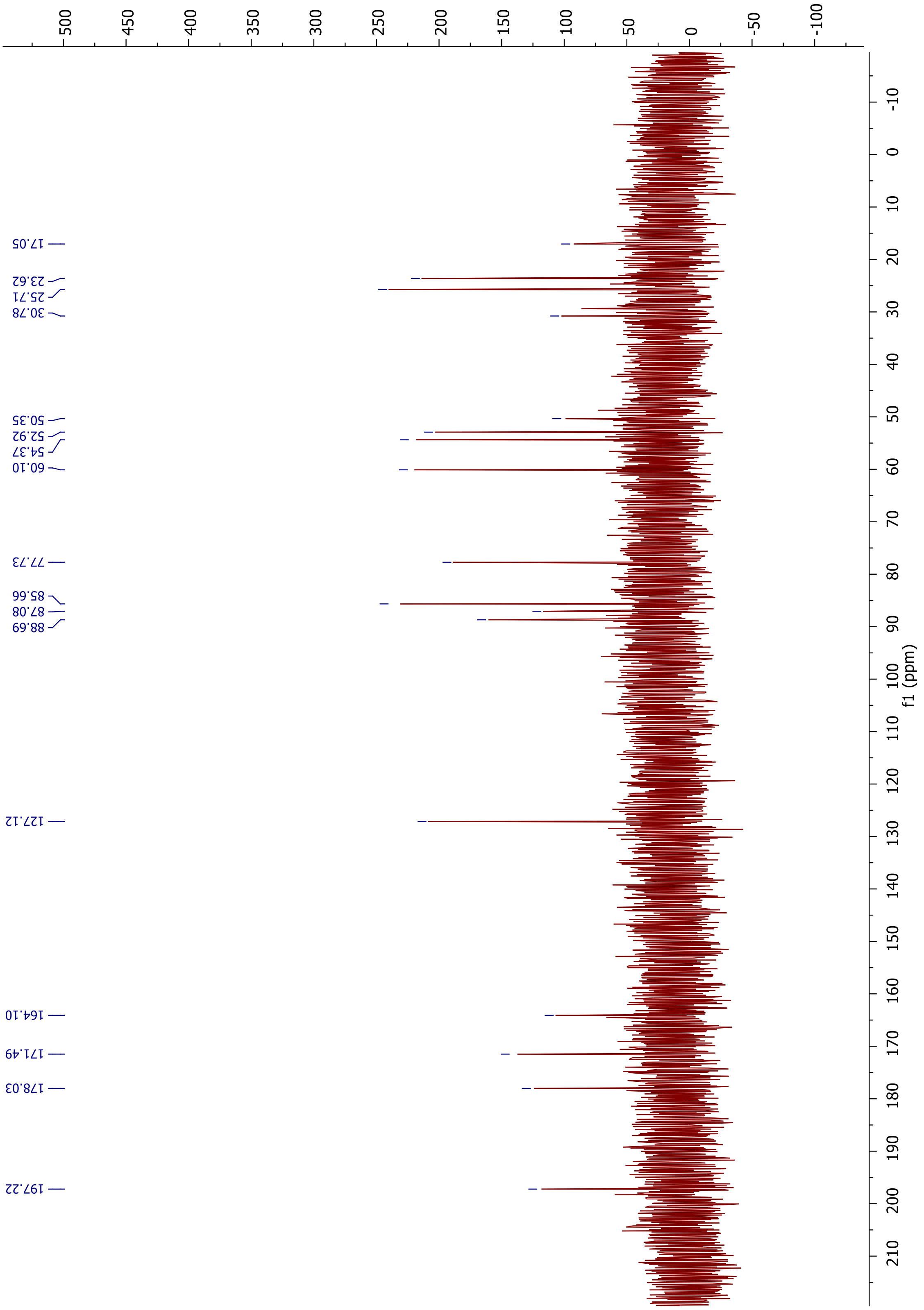

*Figure S 14. ^{13}C-NMR spectrum of **2**.*

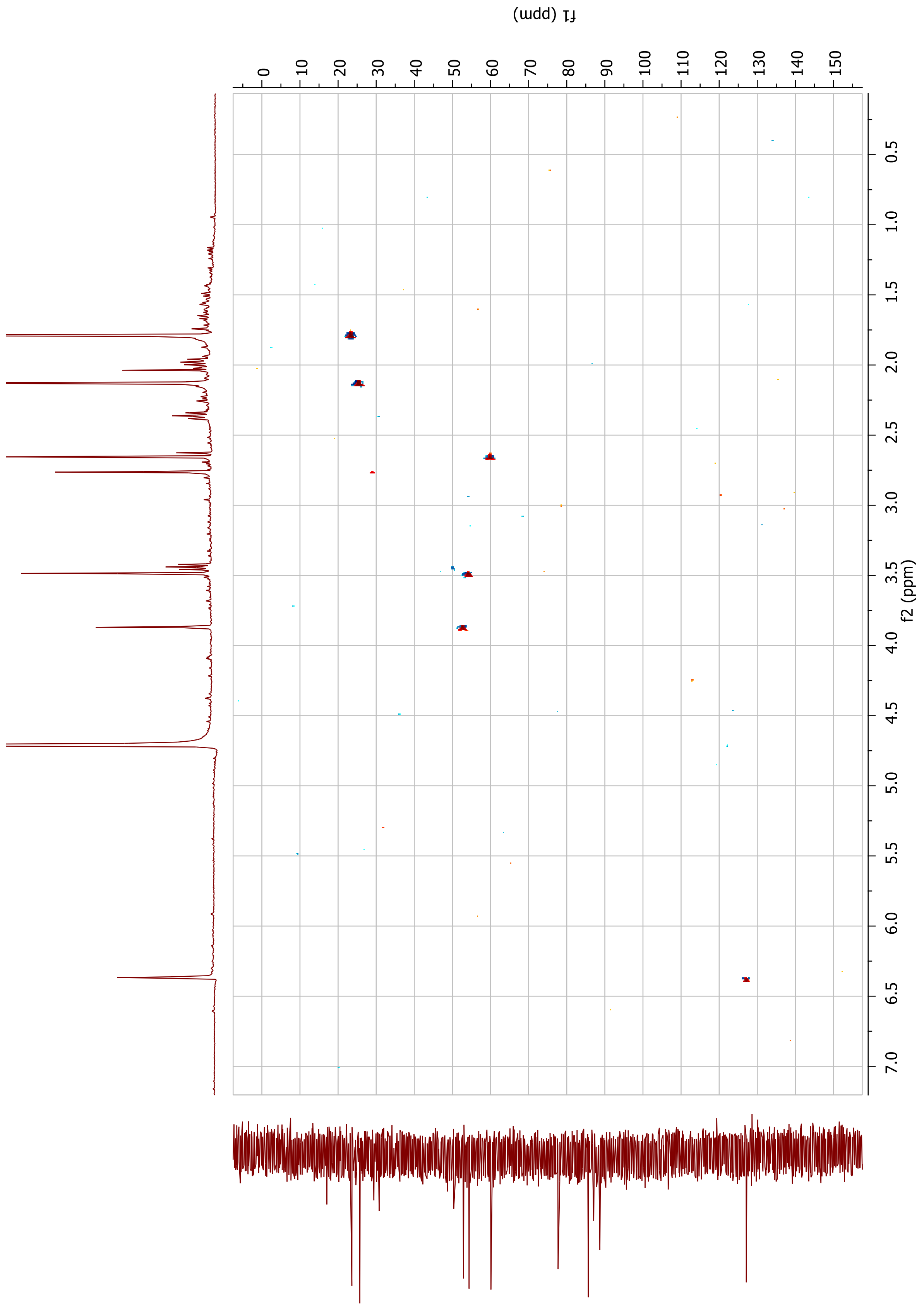

Figure S *15*. HMQC spectrum of **2**.

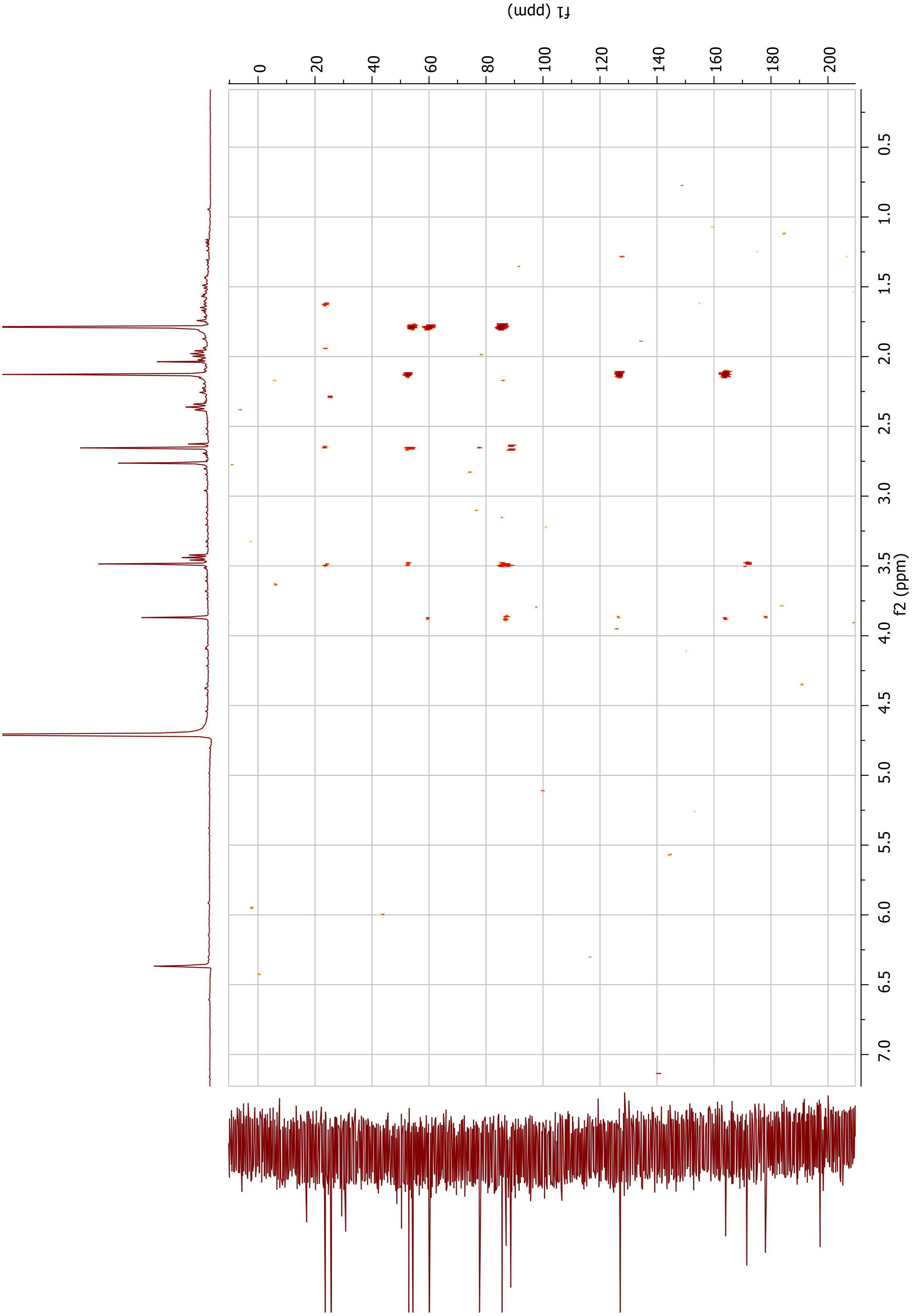

*Figure S 16. HMBC spectrum of **2**.*

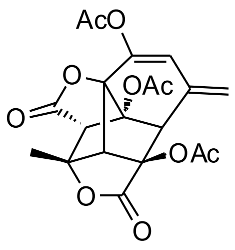

2'

Acetylated dimer 2': To a solution of dimer **2** (0.100 g, 0.32 mmol) in freshly distilled dichloromethane (2 mL) were added Et_3N (0.28 mL, 2.1 mmol) and Ac_2O (0.2 mL, 2.1 mmol). The mixture was stirred overnight at room temperature under a nitrogen atmosphere. After concentration *in vacuo*, water was added (10 mL) and the resulting solution extracted with EtOAc (2x10 mL). The organic layers were pooled, dried over $MgSO_4$, filtrated and concentrated *in vacuo*. A colorless oil was obtained (0.12 g, 90%). **^{1}H NMR** ($CDCl_3$, 400 MHz): δ ppm 6.12 (s, 1H), 5.26 (s, 1H), 5.16 (s, 1H), 3.88 (s, 1H), 3.86 (s, 1H), 3.62 (s, 1H), 2.23 (s, 3H), 2.10 (s, 3H), 2.05 (s, 3H), 1.67 (s, 3H). **^{13}C NMR**: ($CDCl_3$, 100 MHz) δ ppm 170.9 (CO), 169.8 (CO), 169.7 (CO), 169.5 (CO), 169.1 (CO), 140.6 (C), 134.3 (C), 120.9 (C), 88.6(C), 87.3(C), 80.9 (C), 77.7 (C), 58.6 (CH), 55.5 (CH), 50.6 (CH), 23.2 (CH_3), 20.9 (CH_3), 20.7 (CH_3), 20.7 (CH_3), 20,5 (CH_3). **MS *m/z* :** 418(1), 376(20), 334(7), 290(2), 273(3), 266(1), 246(2), 231(3), 214(6), 182(23), 175(3), 162(6), 141(28), 140(100), 91(1), 77(2), 69(2), 43(100).

HRMS: 419.0950, calculated for $[M.H]^+$ $C_{20}H_{19}O_{10}$ 419.0978. Δ= -6,7 ppm.
377.0859, calculated for $[M(-CH_3CO+H).H]^+$ $C_{18}H_{17}O_9$ 377.0873. Δ=-3,7 ppm.
335,0754, calculated for $[M(-2CH_3CO+2H).H]^+$ $C_{16}H_{15}O_8$ 335,0767. Δ=-3,9 ppm.

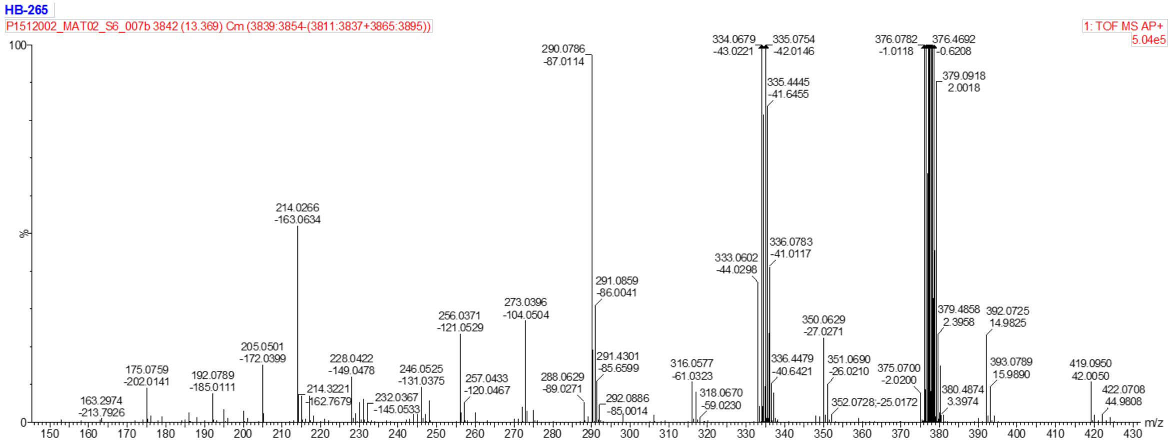

*Figure S 17. HMRS spectrum of **2'**.*

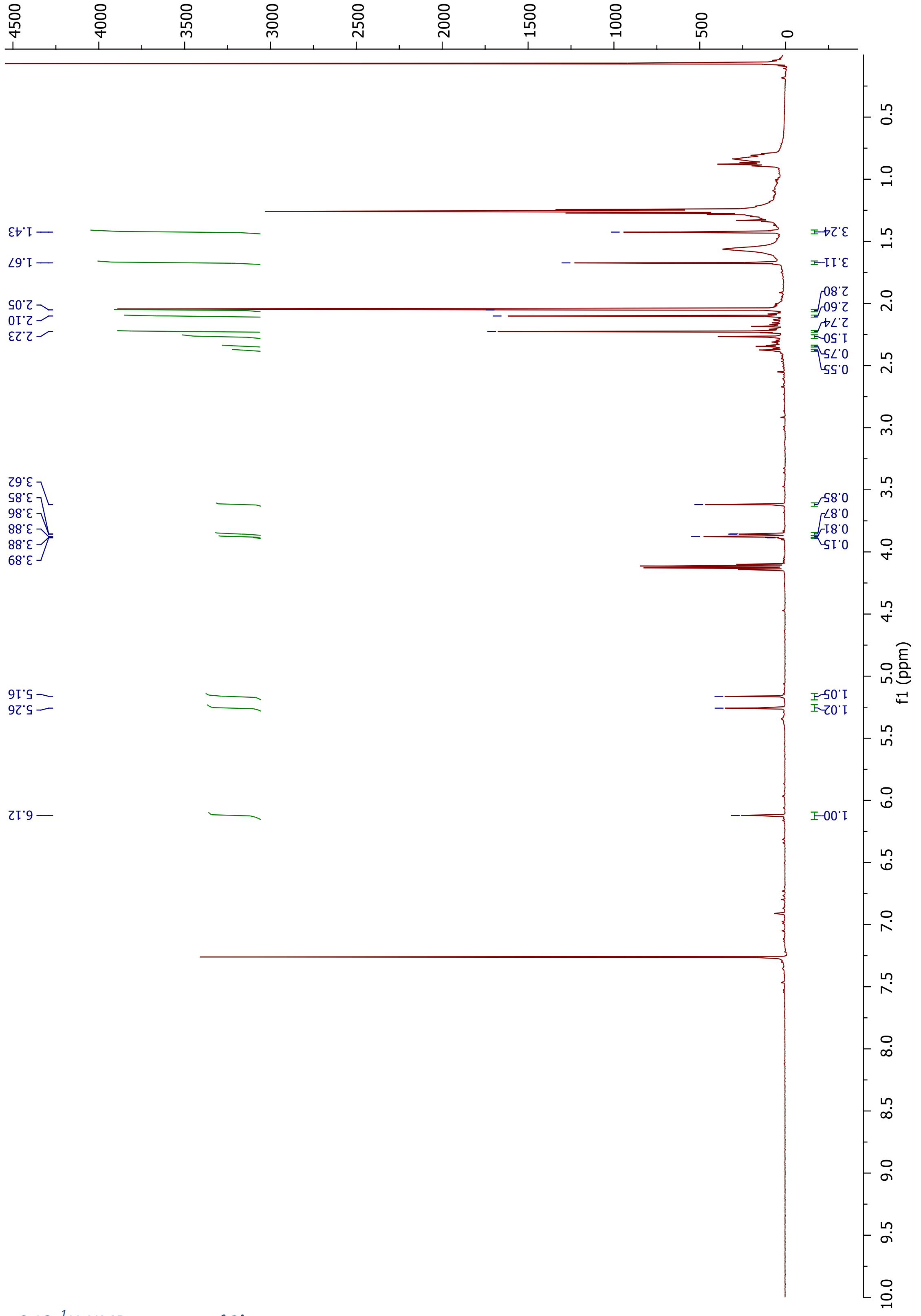

Figure S 18. [1]*H-NMR spectrum of* ***2'****.*

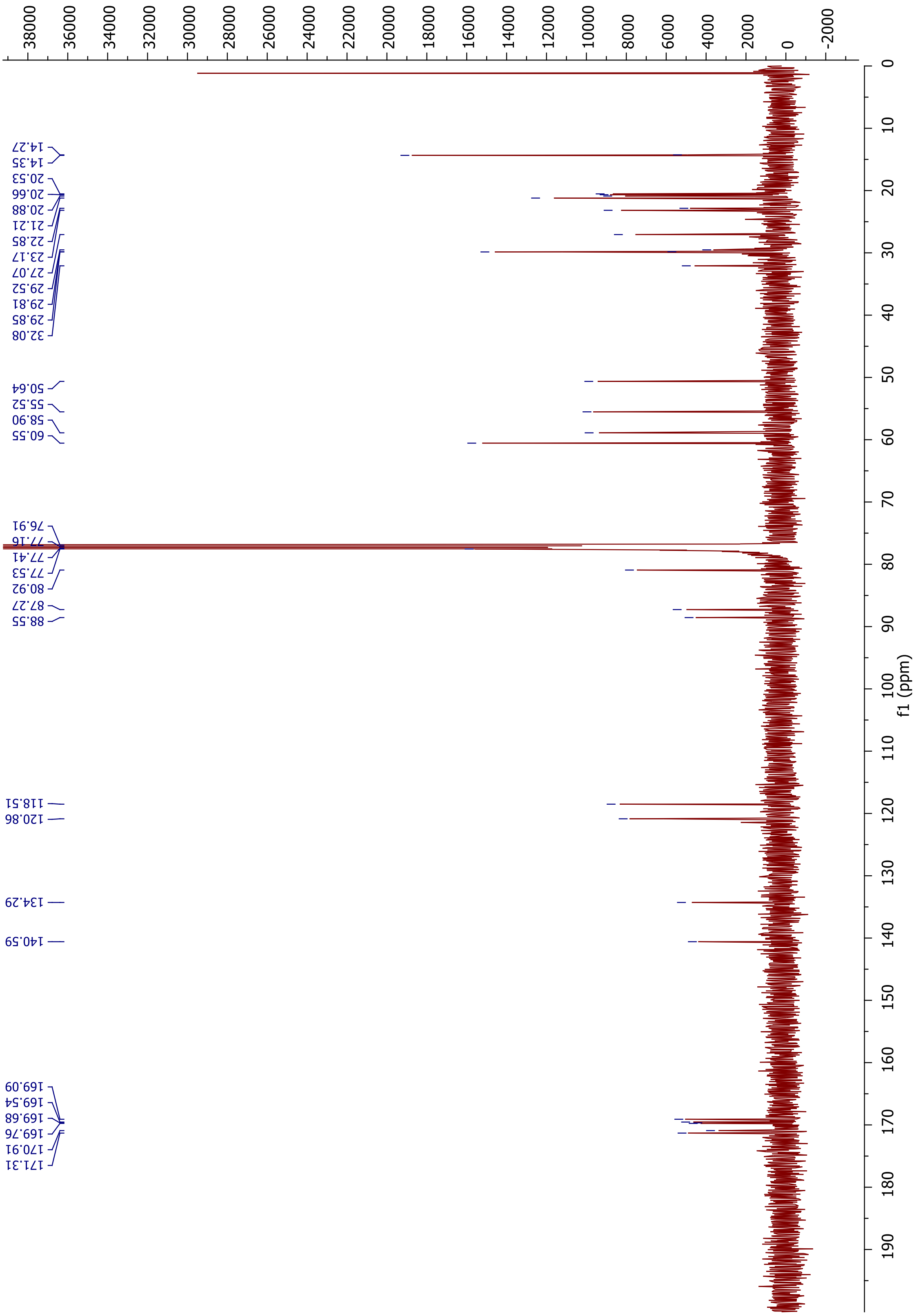

*Figure S 19. ^{13}C-NMR spectrum of **2'**.*

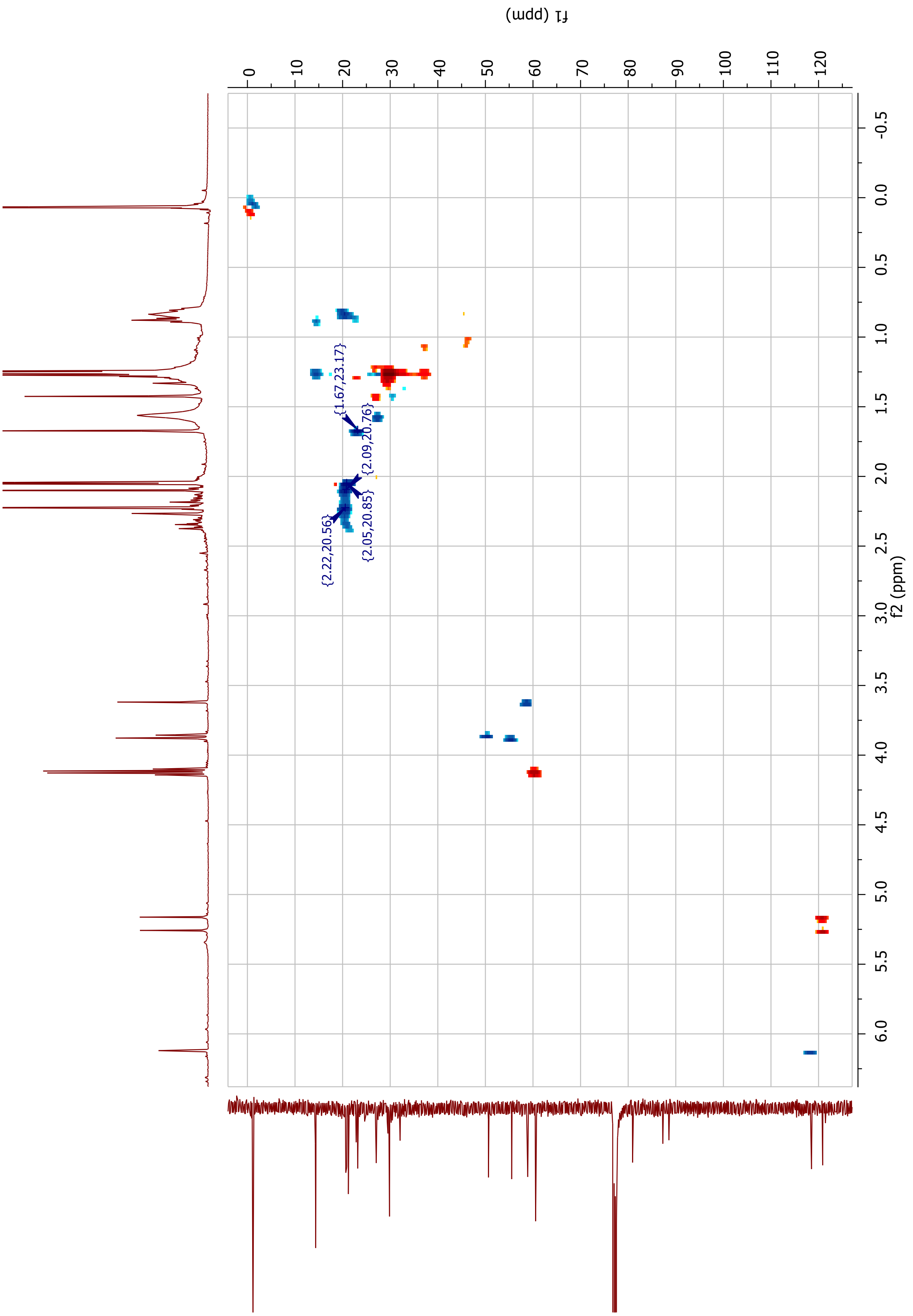

*Figure S 20. HMQC spectrum of **2'**.*

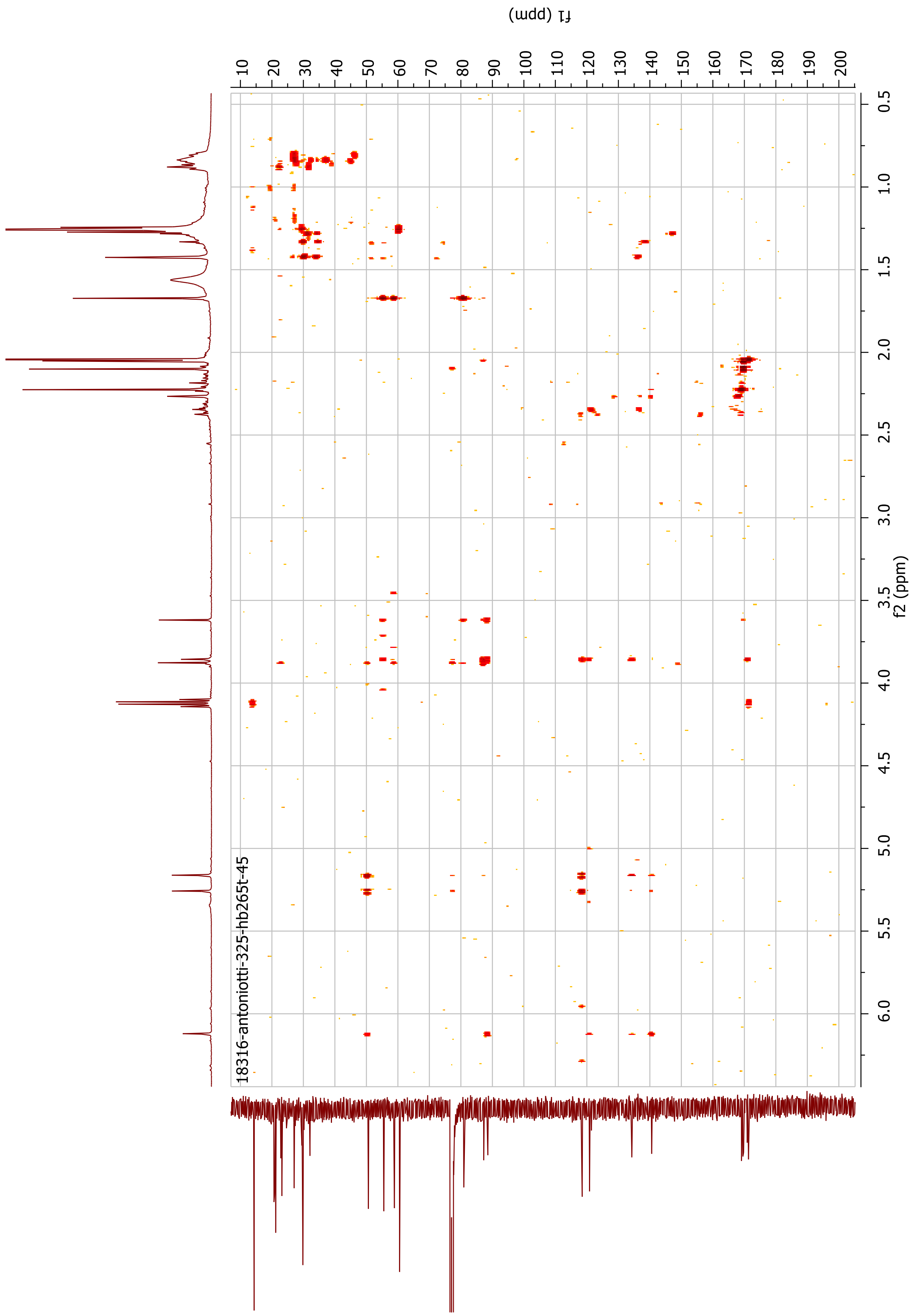

*Figure S 21. HMBC spectrum of **2’**.*

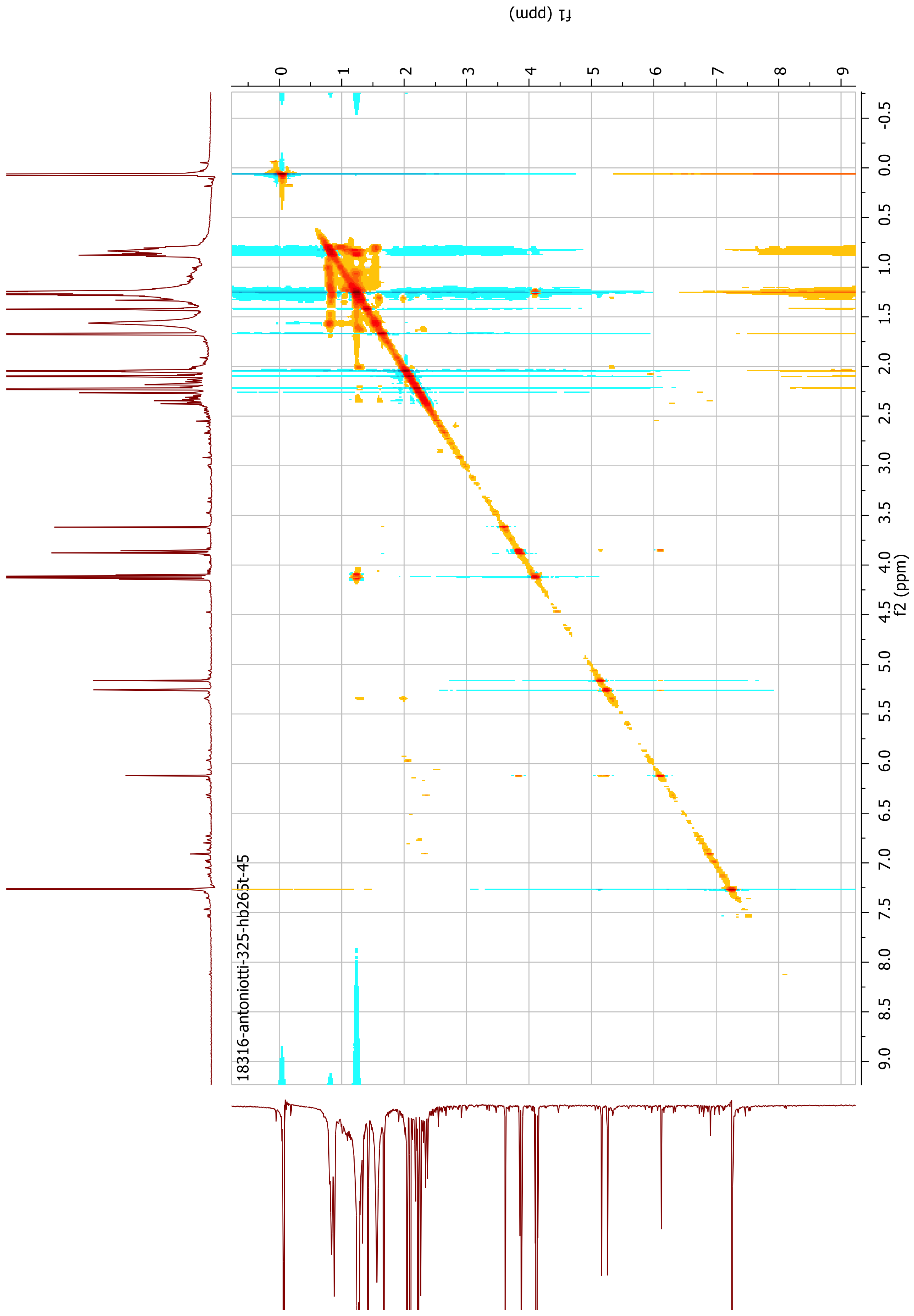

*Figure S 22. COSY of **2’**.*

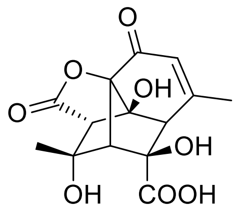

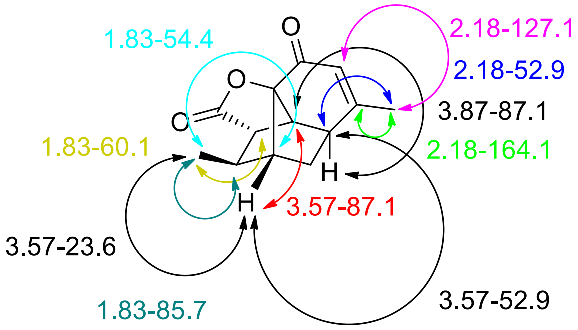

*Figure S 23. HMQC correlation for **2**.*

*Table S 1. ^{1}H and ^{13}C-NMR chemical shifts of **2'** with alternative numbering system.*

	**2'**[a]		
**No.**	δ_C	$\delta_{H,}$ mult. (J in Hz)	HMBC
**1**	140.6	-	
**2**	88.6	-	
**3**	87.3	-	
**4**	50.6	3.86, s	C-3, C-2, C-3', C-2'
**5**	134.3	-	
**6**	118.5	6.12, s	C-1, C-2, C-4, C-5, C-7,
**7**	120.9	5.26, d (2)<br>5.16, d (2)	C-4, C-5, C-6
**1′**	169.8	-	
**2′**	170.9	-	
**3′**	77.7	-	
**4′**	55.5	3.88, s	C-2', C-3', C-5', C-6', C-7', C-3
**5′**	80.9	-	
**6′**	58.9	3.62, s	C-4', C-5', C-2, C-3
**7′**	23.2	1.67, s	C-4', C-5', C-6'
**OAc-1**	169.1	-	
	20.5	2.23, s	
**OAc-3**	169.5	-	
	20.9	2.05, s	
**OAc-3'**	169.7	-	
	20.7	2.10, s	

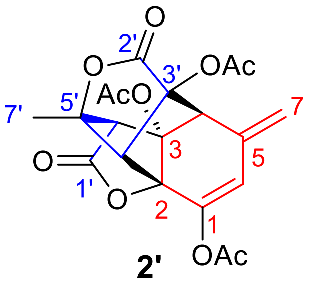

2'

[a] $CDCl_3$, 400 and 100 MHz, respectively, δ ppm.
